# Identification of a novel *Scn3b* mutation in a Chinese Brugada syndrome pedigree: implications for Nav1.5 electrophysiological properties and intracellular distribution of Nav1.5 and Navβ3

**DOI:** 10.3389/fcvm.2024.1320687

**Published:** 2024-02-20

**Authors:** Jun Fan, Shao-hua Wang, Li-li Cao, Wei-jie Li, Shao-xi Sun, Shao-ling Luo, Yi-chao Pan, Wen-liang Tan, Tian-yuan Wu, Zhen Liu, Bing-bo Yu

**Affiliations:** ^1^ Department of Cardiology, Guangzhou First People’s Hospital, School of Medicine, South China University of Technology, Guangzhou, Guangdong, China; ^2^ Department of Otolaryngology-Head & Neck Surgery, Guangdong Second Provincial General Hospital, Guangzhou, China

**Keywords:** Brugada syndrome (BrS), Chinese, SCN3B, mutatation, sodium current

## Abstract

**Background:**

The *Scn3b* gene encodes for Navβ3, a pivotal regulatory subunit of the fast sodium channel in cardiomyocytes. However, its mutation status in the Chinese population suffering from Brugada Syndrome (BrS) has not been characterized, and the contributory pathophysiological mechanisms to disease pathology remain undefined.

**Methods and Results:**

A *Scn3b* (c.260C>T, p.P87l) mutation was identified in a patient with BrS of Chinese descent. Functional analyses demonstrated that sodium channel activation for the wild type, mutant samples, and co-expression of both commenced at −55 mv and peaked at −25 mv. The mutant group exhibited a notable reduction, approximately 60%, in peak sodium channel activation current (I_Na_) at −25 mv. The parameters for half-maximal activation voltages (V_1/2_) and slope factors (k) showed no significant differences when comparing wild type, mutant, and combined expression groups (*P *= 0.98 and *P *= 0.65, respectively). Additionally, no significant disparities were evident in terms of the steady-state sodium channel inactivation parameters V_1/2_ and k (with *P*-values of 0.85 and 0.25, respectively), nor were there significant differences in the activation time constant τ (*P* = 0.59) and late sodium current density (*P* = 0.23) across the wild-type, mutant, and co-expressed groups. Confocal imaging and Western blot analysis demonstrated decreased plasma membrane localization of SCN3B and SCN5A in the P87l group. Computational simulations of cardiac action potentials suggested that SCN3B P87l can alter the morphology of the action potentials within the endocardium and epicardium while reducing the peak of depolarization.

**Conclusions:**

The pathogenic impact of the *Scn3b* P87l mutation predominantly originates from a reduction in peak I_Na_ activation current coupled with decreased cell surface expression of Nav1.5 and Navβ3. These alterations may influence cardiac action potential configurations and contribute to the risk of ventricular arrhythmias in individuals with BrS.

## Introduction

Brugada Syndrome (BrS), a condition associated to sudden cardiac death in patients with no structural heart disease, was initially reported by Brugada in 1992 (1). This syndrome accounts for about half of the sudden deaths in individuals with no structural heart disease and is the leading cause of natural mortality in males under 50 in South Asia (2). As gene sequencing techniques have advanced, primarily with breakthroughs in next-generation sequencing technology, over 40 mutations associated with ion channels have been proposed to be associated with BrS (3). Although mutations in *SCN5A* account for 11%–28% of BrS cases, most genetic mutations associated with this syndrome occur with a frequency of less than 1% (4). The relationship between these mutations and BrS development necessitates further validation. Moreover, nearly 60% of individuals diagnosed with BrS do not have a specific identifiable genetic mutation (5). This suggests that the actual genetic landscape underpinning BrS may be more complex than initially perceived.

Recent research indicates that variants in the *Scn3b* gene, responsible for encoding a regulatory subunit of the fast sodium channel, could be implicated in BrS (6). However, the association between mutations in the *Scn3b* gene and BrS has not been established in the Chinese cohort. Furthermore, the pathogenic effects of *Scn3b* mutations in BrS patients are not yet fully understood. Consequently, there is a need for additional research to clarify the role of *Scn3b* gene variations in the manifestation of BrS within the Chinese population.

## Methods

### Diagnostic criteria for BrS

The definition of BrS adheres to the criteria delineated in the 2022 ESC (European Society of Cardiology) Guidelines for the management of patients with ventricular arrhythmias and the prevention of sudden cardiac death (7). According to these guidelines, a diagnosis of BrS should be established in the following contexts: (1) When patients present with a spontaneous type 1 Brugada Electrocardiogram (ECG) pattern and have no other coexisting heart disease, BrS diagnosis is recommended. (2) In patients who have survived a cardiac arrest (CA) resulting from either ventricular fibrillation (VF) or polymorphic ventricular tachycardia (PVT), and exhibit a type 1 Brugada ECG pattern elicited through a sodium channel blocker challenge or during episodes of fever, a BrS diagnosis is advised—assuming no other heart disease is present. Additionally, consideration for a BrS diagnosis is warranted in patients with no other cardiac pathology who display an induced type 1 Brugada ECG pattern and have at least one of the following risk factors: (1) A history of arrhythmic syncope or nocturnal agonal respiration. (2) A familial history of BrS. (3) A family history of Sudden Death at an age younger than 45 years where autopsy findings were negative and circumstances arouse suspicion of BrS. (4) Furthermore, in the absence of other heart diseases, the diagnosis of BrS may also be contemplated for patients who demonstrate an induced type 1 Brugada ECG.

### Ethical compliance

The Ethics Committee of the Second Affiliated Hospital of South China University of Technology has granted approval for the conduct of this study. The privacy and confidentiality of the participants were respected and protected throughout the research process, and all aspects of the study complied with the principles of the Declaration of Helsinki.

### Family pedigree analysis

The patient's family history was detailed, disclosing a living mother but a deceased father. In addition to the patient, the family unit comprises one brother, one sister, and a son. To investigate the genomic landscape, comprehensive sequencing was performed on blood samples obtained from all accessible family members. Conjointly, ECG analyses were executed to screen for cardiac anomalies. For family members whose ECGs did not exhibit a Brugada pattern at baseline, a procainamide provocation test was administered to unmask any latent aberrations.

### Identification of suspected pathogenic genes

To determine the possible disease-causing genetic mutation in the patient, whole exome sequencing was performed on blood samples at the Beijing Genomics Institute, China. A report listing the genetic mutations was generated, indicating several potential candidate genes for the condition. The details of these suspect pathogenic genes are presented in Supplementary Table S1.

Suspected pathogenic gene variants were assessed in accordance with the “Standards and Guidelines for the Interpretation of Sequence Variants: a joint consensus recommendation by the American College of Medical Genetics and Genomics and the Association for Molecular Pathology” (8). Briefly, these guidelines categorize genetic mutations' potential pathogenicity into four tiers: Pathogenic, Likely Pathogenic, Benign, and Likely Benign. The preliminary screening process for the suspect genes involved two key steps: Initially, the genes were subjected to preliminary assessment using the guidelines' “Criteria for Classifying Pathogenic Variants” (Supplementary Table S2) and the “Criteria for Classifying Benign Variants” (Supplementary Table S3). Subsequently, the “Rules for Combining Criteria to Classify Sequence Variants” (Supplementary Table S4) were applied to evaluate and quantify the likelihood of the gene mutations being pathogenic. In addition to our *in vitro* experimental validation, and adhering to the aforementioned criteria, the SCN3B (NM_018400, c.260C>T, pP87l) mutation emerges as the variant with the most substantive evidence indicating its pathogenic potential.

### Mutagenesis, cell culture and transfection

Expression plasmids for both the wild-type and P87l mutated *Scn3b* and *Scn5a* were generated. Nav1.5 cDNA (also recognized as *Scn5a*, NM_198056) was subcloned into the mammalian expression vector pENTER, while *Scn3b* cDNA (NM_018400) was amplified via PCR and then subcloned into the pIRES2-EGFP vector. The P87l mutations were induced using site-directed mutagenesis with the QuikChange II kit (Stratagene, CA, USA). Additionally, *Scn3b* cDNA (NM_018400) was amplified and subcloned into the pEGFP-N1 vector, which expresses a fusion of Navβ3 and eGFP (Enhanced Green Fluorescent Protein). This permits easy visualization of the intracellular distribution of Navβ3.

HEK293 cells were obtained from the Shanghai Institute of Cell Research, Chinese Academy of Sciences and cultured in Dulbecco's Modified Eagle Medium (DMEM) enriched with 10% fetal bovine serum. Cultivation conditions were maintained at a constant 37°C and 5% CO_2_ atmosphere. The cells were split in a 1:3 ratio upon reaching 90% confluence, employing a previously outlined method. Upon achieving 70% confluence, HEK293 cells were transfected with *Scn5a* and *Scn3b* plasmids at equimolar concentrations by following the manufacturer's protocol for Lipofectamine 3,000 (Invitrogen; Thermo Fisher Scientific, Inc.).

For Western blot analysis, HEK293 cells were transfected with plasmids encoding the Nav1.5 protein and its auxiliary subunit Navβ3, either in wild-type or mutant forms. For electrophysiological studies, the transfected HEK293 cells were categorized into three groups based on their expression profile: one group expressing Nav1.5 with wild-type Navβ3, a second group with Nav1.5 and mutant Navβ3, and a third group with Nav1.5 co-expressing both wild-type and mutant Navβ3 at a 1:1 molar ratio, keeping the total quantity consistent with the prior groups. Additionally, for confocal microscopy assays, cells were transfected with plasmids encoding a Navβ3-eGFP fusion protein to study the localization and distribution of this subunit within the cellular environment.

### Western blotting

Membrane proteins and cytoplasmic proteins were extracted using the Membrane and Cytosol Protein Extraction Kit (Beyotime, China). Protein concentrations in the samples were balanced using the bicinchoninic acid (BCA) method. The samples were then subjected to sodium dodecyl sulfate polyacrylamide gel electrophoresis (SDS-PAGE). A polyvinylidene fluoride (PVDF) membrane was blocked with 5% skim milk at room temperature for 1 h. Primary antibodies for Navβ3 (Cat No: #27368, SAB, Inc.), Nav1.5 (Cat No: #33488, SAB, Inc.), GADPH (Cat No. 10494-1-AP, Proteintech Group, Inc.), and ATP1A2 (Cat No. 16836-1-AP, Proteintech Group, Inc.) were diluted in 5% skim milk as per the instruction manual, at ratios ranging from 1:1,000 to 1:5,000. The PVDF membrane was then incubated overnight with the appropriate primary antibodies on a shaker at 4°C. After this, the PVDF membrane was washed three times with phosphate buffered saline with tween-20 (PBST). Horseradish peroxidase (HRP)-conjugated anti-rabbit or anti-mouse antibodies were diluted at ratios ranging from 1:50,000 to 1:10,000. The PVDF membrane was subsequently treated with the secondary antibody in a shaker for one hour at room temperature. Proteins were then detected using an enhanced chemiluminescence method and captured on x-ray film in a dark setting.

### Cell immunofluorescence

A confocal culture dish containing transfected cells was treated with 1,1'-dioctadecyl-3,3,3′,3′-tetramethylindocarbocyanine perchlorate (Dil, 3 μm, D4010, US Everbright, Inc.) to stain the cellular membrane. Following this, the cells were fixed using a 4% paraformaldehyde solution at room temperature for a duration of 30 min. The cells were then exposed to 4′,6-diamidino-2-phenylindole (DAPI, 1 μg/ml, Invitrogen; Thermo Fisher Scientific, Inc.) for 5 min. Lastly, an appropriate volume of PBS buffer solution was introduced, and the cellular observations were made using a Zeiss LSM780 microscope.

Cells expressing eGFP and adhering to the dish were identified using a low power lens under light with an excitation wavelength of 488 nm. Subsequent observation of subcellular structures was carried out using an oil immersion microscope with a magnification of 630 times. The distribution of Dil was visualized by illuminating it with a laser excitation source at a wavelength of 549 nm. The distribution of DAPI was observed through excitation with a laser beam of 360 nm wavelength. Lastly, the distribution of the fusion protein eGFP-SCN3B was examined utilizing a laser source with an excitation wavelength of 488 nm.

### Electrophysiological assays

The solutions needed for whole cell patch clamp experiments were prepared as follows: The bath solution comprised of 140 mm NaCl, 4 mM KCl, 1 mm MgCl_2_, 2 mm CaCl_2_, 5 mm D-glucose monohydrate, and 10 mm HEPES, with the pH calibrated to 7.4 using NaOH. The pipette solution contained 145 mm CsCl, 0.1 mm CaCl_2_, 2 mm MgCl_2_, 10 mm NaCl, 0.5 mm Na_2_-GTP, 2 mm Mg-ATP, 1.1 mm EGTA, and 10 mm HEPES, with the pH adjusted to 7.2 with CsOH. The glass electrode was prepared using a P-97 puller from Sutter, USA, as detailed in our prior study. Once the electrode was filled with the solution, its resistance measured between 1-3MΩ. Signals were captured at a sampling rate of 10 kHz and filtered at 2 kHz. The cellular current was recorded in the whole-cell patch-clamp mode. A HEKA USB-10 amplifier facilitated the elicitation of currents through pulse protocols, while the PatchMaster software was employed to record the currents from HEK293 cells.

The sodium channel current was recorded using the voltage clamp mode. Following this, a series of curves, including the voltage-current (IV) curve, activation curve, depolarization curve, re-covery curve, and late sodium current were plotted. The stable activation curve of sodium channels was documented by plotting the pulse voltage against the whole cell conductance. Here, conductance (G) was calculated as the peak value of the whole cell current divided by the difference between the depolarizing pulse voltage (V_m_) and the channel reversal potential (V_rev_). Both activation and inactivation curves were fitted using the Boltzmann equation, G/g_max _= 1/(1 + exp[(V_m_−V_1/2_)/k]); where G represents activation conductance, g_max_ the maximum activation conductance, V_1/2_ the pulse voltage at 50% activation, and k is the slope factor. The reactivation curve was fit using an exponential function: I/I_max _= A + A1 × exp(-t/τ), where t denotes time and τ is the recovery time constant. For all data processing and analysis, the Origin 11 software (OriginLab Corporation) was utilized.

### Simulation of action potential (AP)

To explore the properties of AP in the endocardium and epicardium, we utilized a human ventricular cell model formulated by Tomek and Jakub. This model, which was accessible via https://models.physiomeproject.org/workspace/5e1, sets a duration of 1 millisecond (ms) for the initiation of a cardiac myocyte's action potential.

### Statistical analysis

Data processing and statistical analysis across the entire dataset were conducted using R, a language and environment for statistical computing (R Foundation for Statistical Computing, Vienna, Austria, version 4.0.2). Continuous random variables were presented as mean ± standard deviation or mean ± standard error of the mean. When comparing two distinct groups, the *t*-test was employed for distributions that were both normal and had equal variances. In cases where these conditions were not met, analysis of variance (ANOVA) was utilized instead. For the evaluation of the sodium channel current, including the peak I–V relationship, steady-state activation, inactivation, reactivation parameters, and late currents, we applied a 2-way ANOVA coupled with Tukey's Honestly Significant Difference (HSD) test to identify statistical significance among the multiple comparisons. A key aspect of this research focused on the activation of sodium channels, which commences at −50 mv. Hence, we meticulously computed the exact *p*-values for all voltage steps spanning from −55 mv to 50 mv to to evaluate the activation properties of the sodium channels. Throughout our analysis, we adopted a *P*-value threshold of 0.05, below which results were considered to be statistically significant.

## Results

### The clinical characteristics of the BrS patient

**Figure 1:**
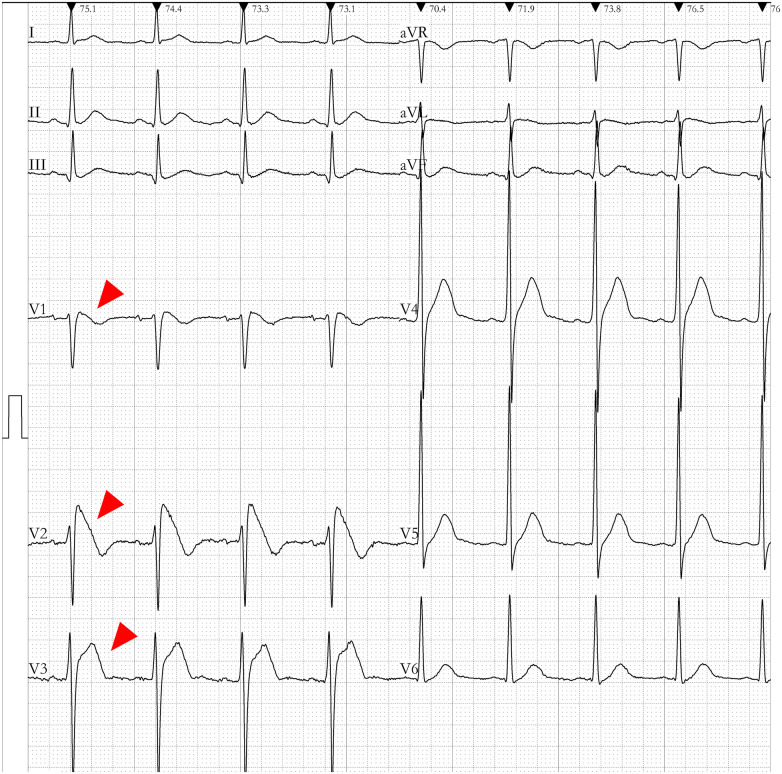
Electrocardiogram (ECG) of the enrolled brugada syndrome (BrS) patient. The ECG of the patient demonstrated ST-segment elevation in the anterior lead (highlighted by the red arrow). The V_1_–V_2_ leads presented a downsloping ST-segment elevation, while the V_5_–V_6_ leads did not exhibit the typical pattern of a right bundle branch block.

Data was gathered pertaining to a patient who survived an incident of sudden cardiac death. On the 10th of August, 2017, the patient experienced an unexpected loss of consciousness with no overt precipitating factors. The electrocardiographic monitor recorded an onset of ventricular fibrillation in the patient. The patient's electrocardiography (ECG) exhibited a Type I Brugada pattern, as elucidated in Figure 1. A comprehensive structural assessment of the heart, which included ultrasonography and coronary angiography, indicated an absence of structural heart diseases. Taking into consideration the patient's documented history of ventricular fibrillation, the morphology of the ECG, and the absence of detectable structural heart abnormalities, a diagnosis of BrS was made. While we had implanted an Implantable Cardioverter Defibrillator (ICD) for the individual, it had been depleted of power for some time. Due to financial constraints, the ICD was not replaced, and the patient subsequently suffered a sudden cardiac death outside of the hospital setting and could not be resuscitated.

**Figure 2:**
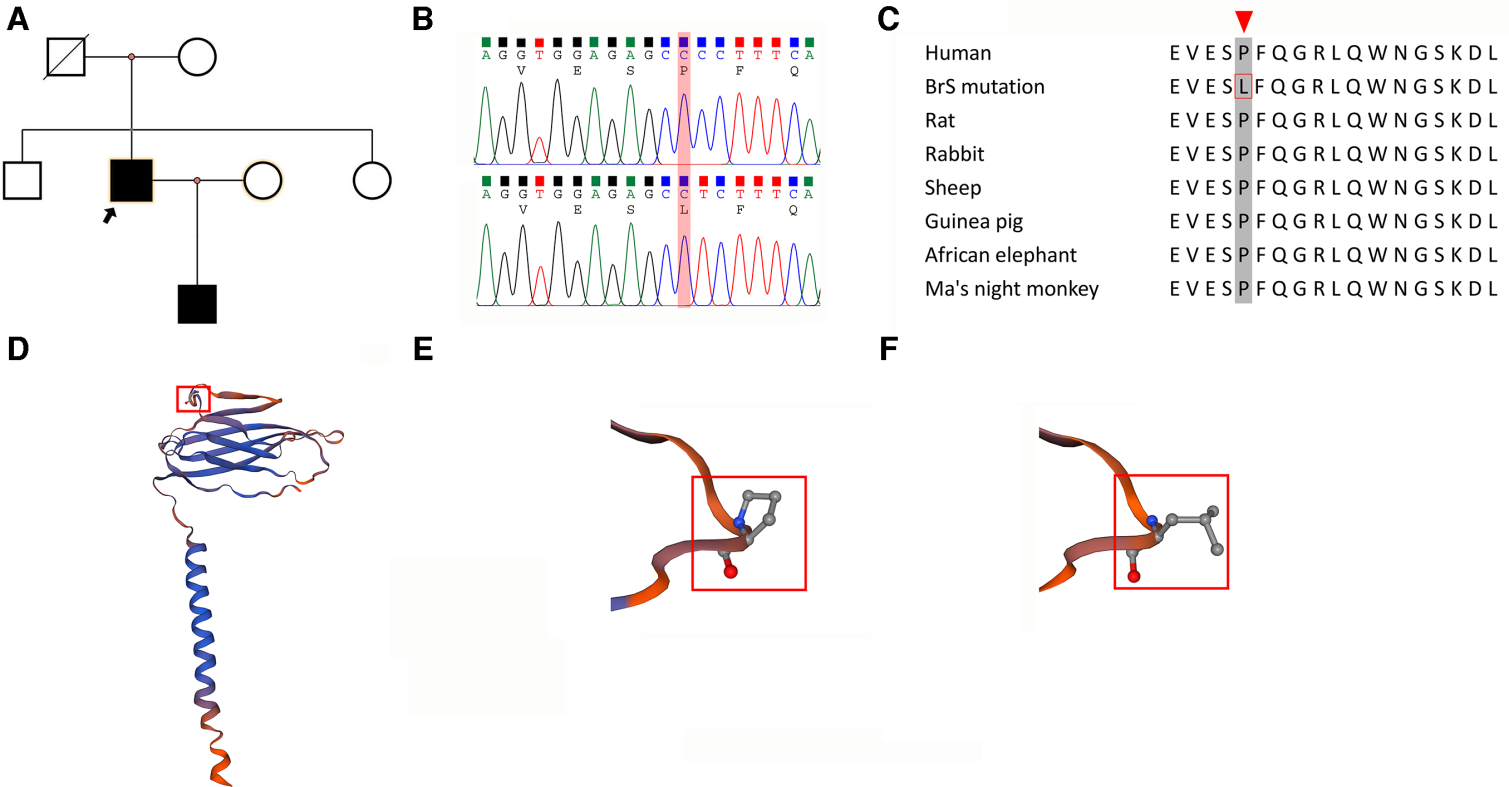
Characterization of the SCN3B P87l mutation. (**A**) Pedigree of a Family with Brugada syndrome: The pedigree illustrates the inheritance of Brugada syndrome within a family. Squares indicate male members, and circles represent female members. The proband is indicated with a black arrow. Black-filled symbols denote individuals diagnosed with Brugada syndrome, while blank symbols indicate unaffected members. Deceased individuals with unknown electrocardiogram status are marked by a slash. (**B**) Comparison of Wild-Type and Mutant SCN3B Nucleotide Sequences: The sequence alignment shows a cytosine-to-thymine substitution at nucleotide position 260 in exon 3 of SCN3B (P87l mutation). This mutation leads to a change from proline to leucine at the protein level. (**C**) Conservation of Proline at Position 87 Across Species: An alignment of SCN3B amino acid sequences from various mammalian species highlights the conservation of the proline residue at position 87, as indicated by a red arrow. (**D**) Molecular Model of Navβ3: Utilizing the SWISS-MODEL, the molecular structure of Navβ3, which is encoded by the SCN3B gene, has been depicted. It shows a membrane-associated protein with a single transmembrane domain. The proline at position 87 is prominent in the extracellular segment and is marked in red. (**E**) Hydrophilicity of Proline at Position 87: The proline residue at position 87 in the Navβ3 protein is characterized as hydrophilic. (**F**) Transition to Hydrophobicity in the Mutant: In the P87l mutant form of SCN3B, the 87th amino acid shifts from the hydrophilic proline to the hydrophobic leucine.

The pedigree of the patient is depicted in Figure 2A. The clinical investigations revealed that the patient's mother did not exhibit a Brugada pattern on her ECG, and the procainamide challenge did not induce such a pattern. Furthermore, genomic sequencing indicated the absence of the *Scn3b* mutation in her. The patient's siblings also displayed normal ECGs, with no Brugada patterns induced by the drug challenge, and genetic sequencing confirmed they did not carry the *Scn3b* mutation. Interestingly, the patient's son exhibited a Brugada pattern on his ECG (Supplementary Figure S1) without drug provocation.

### Screening for suspected pathogenic genes

In pursuit of identifying causative genetic mutations associated with BrS, a hereditary condition, blood samples were collected and subjected to whole exome sequencing at the Beijing Genomics Institute. The sequencing process utilized a Nimblegen full exome capture chip, followed by comprehensive analysis using an Illumina Hiseq sequencer, ensuring a target sequence coverage of at least 99%.

The variants that were suspected to be pathogenic underwent rigorous assessment based on the “Standards and Guidelines for the Interpretation of Sequence Variants” established by the American College of Medical Genetics and Genomics in conjunction with the Association for Molecular Pathology, as previously described in the methods section (8). Integrating our *in vitro* validation of the SCN3B variant's impact on sodium currents, the *Scn3b* P87l (c.260C>T) mutation (Figure 2B) was adjudicated as Likely Pathogenic. The detailed justification for this categorization is provided in Supplementary Tables S5–S7. Materials pertaining to the determination of the pathogenicity of the gene mutations are provided in Supplementary Presentation S1, while the computational predictions of the gene mutations' pathogenicity can be found in Supplementary Presentation S1. Other detected variants (Supplementary Table S1) were evaluated and classified as Benign or Likely Benign, with the specific criteria for these determinations outlined in Supplementary Presentation S1.

A reference sequence comparison highlighted the conservation of amino acid residue 87 in the SCN3B across humans and various mammalian species, as depicted in Figure 2C. Using the Swiss model, we constructed a structural model of Navβ3 encoded by the *Scn3b* gene that revealed its single transmembrane architecture, shown in Figure 2D. Further structural analysis indicated that the P87l substitution—replacement of proline with leucine at position 87—led to a notable conformational alteration of the protein, which is detailed in Figures 2E,F.

### Implications of *SCN3B* P87l on Navβ3 Localization

**Figure 3:**
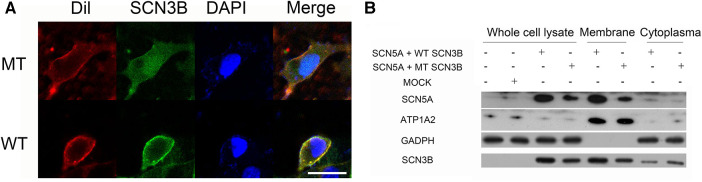
Comparison of WT and P87l Navβ3 protein localization in HEK293 cells. (**A**) HEK293 cell membranes were labeled with red Dil fluorescence. Navβ3 locations were tracked with green fluorescence using WT/P87l eGFP-Navβ3 fusion proteins; DAPI, a nuclear marker, displayed with blue fluorescence. Image analysis showed reduced membrane expression of Navβ3 and increased intracellular expression in P87l samples compared to WT. Scale: 20 μm. (**B**) Nav1.5 and Navβ3 proteins were expressed in HEK293 cells, with GAPDH and ATP1A2 used as references for whole cell lysate/cytoplasmic and membrane components respectively. Both proteins were majorly membrane-bound, with minimal cytoplasmic presence. However, P87l samples revealed less Nav1.5 and Navβ3 on the membrane, but more in the cytoplasm, compared to WT.

We examined the localization of Navβ3 protein in HEK293 cells transfected with *Scn3b*-expressing plasmid through the utilization of confocal fluorescence microscopy (Figure 3A). Following its synthesis, Navβ3 was found to be primarily confined to the cell membrane. The Wild-type (WT) and P87l variants of Navβ3 proteins exhibited green fluorescence and were predominantly detected along the cell membrane with minimal presence in the nucleus and slightly increased expression in the cytoplasm. These observations suggest that Navβ3 primarily functions as a membrane protein. Both WT and P87l Navβ3 were capable of being transported to the cell membrane, albeit with the fluorescence intensity of the P87l variant being relatively muted compared to the WT Navβ3 on the cell membrane. A nominal expression was observed in the cytoplasm for both P87l and WT Navβ3, with the P87l Navβ3 exhibiting a elevated level of expression.

### Influence of P87l on protein expression levels

The study transfected HEK293 cells with *Scn3b*-WT/*Scn3b*-P87l/pcDNA3.1 and *SCN5A* expression plasmid and centered attention on the localization and expression patterns of Navβ3 and Nav1.5 across different cellular compartments including whole cells, cell membranes, and cytoplasm. The results indicated that the SCN3B P87l led to a reduction in Nav1.5 expression at both the total cellular and membranous levels, as illustrated in Figure 3B. Additionally, SCN3B P87l was found to decrease the expression of Navβ3 at both whole cell and membrane protein levels, while concurrently increasing its expression within the cytoplasm.

### Effect of P87l on sodium current (I_Na_)

**Figure 4:**
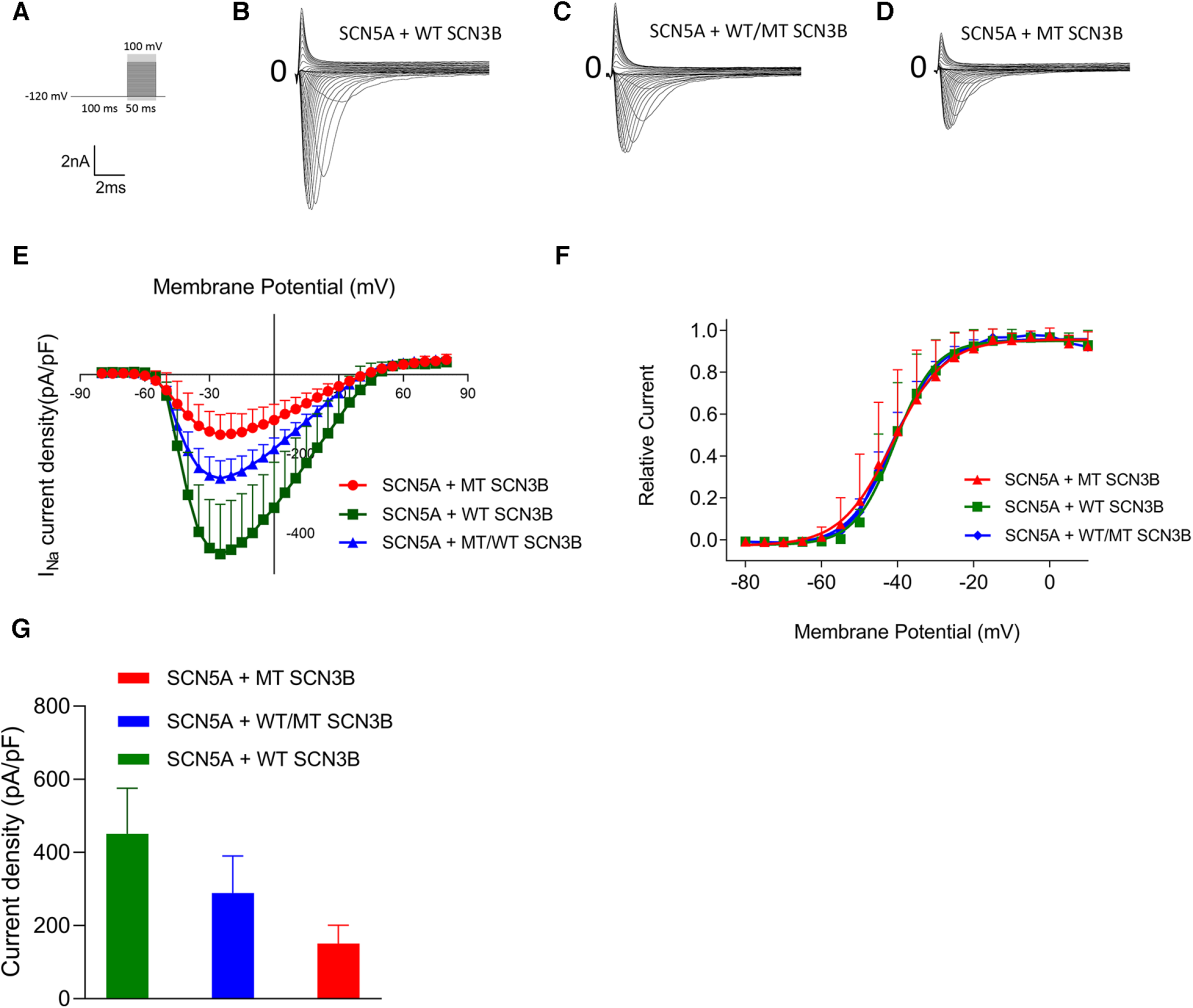
Impact of SCN3B P87l mutation on Nav1.5 sodium channel activation properties. (**A**) The upper panel displays the single-channel activation stimulation protocol, while the lower panel provides the corresponding scale bar. (**B–D**) Representative traces of sodium current (INa) activation are illustrated for the wild-type (WT), mutant (P87l), and co-expression of WT and mutant groups, respectively. (**E**) Activation current-voltage (I–V) curves for the WT, mutant, and co-expressed groups were derived by normalizing currents to cell membrane capacitance. (**F**) Steady-state activation curves of the sodium channels for the WT, mutant, and co-expressed groups are shown. Curves were fitted using the Boltzmann function, revealing no significant differences in the voltage of half-maximal activation (V_1/2_) or slope factor (k) between WT and P87l groups. Refer to Table 1 for detailed statistics. (**G**) The sodium channels in the WT, mutant, and co-expressed groups all reached maximal activation current at a stimulation voltage of −25 mv. A bar graph compares the activation current density at −25 mv for the WT, mutant, and co-expressed groups. Significant differences were found among the groups (*p* = 0.000019).

The parameters for the activation, inactivation, and recovery of the sodium ion channel are summarized in Tables 1–3, respectively. Activation protocols applied to HEK293 cells are depicted in the upper panel of Figure 4A. It was observed that sodium activation currents were induced in the wild-type, mutant, and co-expression of wild-type and mutant groups, as shown in Figures 4A–C, respectively. Sodium channel activation in all groups began at −55 mv and reached maximal activation currents at −25 mv. Specifically, the peak activation currents density at −25 mv for the wild-type, mutant, and co-expressed groups were −450.7 ± 124.9pA/pF, −150.8 ± 50.2pA/pF, and −288.8 ± 101.1/pF (*p* = 0.000019), respectively, as shown in Figure 4G and Supplementary Table S8. The reversal potential for all groups was +50 mv. The differences in maximal activation currents across stimulus voltages ranging from −55 mv to +50 mv for the three groups are detailed in Supplementary Table S8.

**Table 1:** Steady-state activation parameters of sodium channels in HEK 293 cells co-expressing SCN5A with either wild-type (WT), mutant (MT), or a combination of WT and MT SCN3B.

Group	*n*	V_1/2_(mV)					k				
		Value	*P*	*P*_WT-MT_	*P*_WT-WT/MT_	*P*_MT-WT/MT_	Value	*P*	*P*_WT-MT_	*P*_WT-WT/MT_	*P*_MT-WT/MT_
SCN5A + WT SCN3B	8	−40.13 ± 4.98	0.98	0.98	0.99	1	4.71 ± 1.19	0.65	0.62	0.91	0.96
SCN5A + MT SCN3B	8	−40.77 ± 7.46					5.38 ± 1.42				
SCN5A + WT/MT SCN3B	8	−40.60 ± 5.96					5.01 ± 1.35				

The results are expressed as mean ± standard deviation. V_1/2_, voltage of half-maximal activation or inactivation; k, slope factor; WT, wild-type; MT, mutant type; WT/MT, co-expression of wild-type and mutant; WT-MT, comparison between wild-type and mutant groups; WT-WT/MT, comparison between wild-type group and co-expressing group; MT-WT/MT, comparison between mutant group and co-expressing group; *P*, statistical significance value.

**Table 2:** Steady-state in-activation parameters of sodium channels in HEK 293 cells co-expressing SCN5A with either wild-type (WT), mutant (MT), or a combination of WT and MT SCN3B.

Group	*n*	V_1/2_(mV)					k				
		Value	*P*	*P*_WT-MT_	*P*_WT-WT/MT_	*P*_MT-WT/MT_	Value	*P*	*P*_WT-MT_	*P*_WT-WT/MT_	*P*_MT-WT/MT_
SCN5A + WT SCN3B	8	−77.98 ± 2.74	0.10	0.08	0.38	0.64	5.02 ± 0.55	0.25	0.87	0.23	0.47
SCN5A + MT SCN3B	8	−82.94 ± 4.58					4.54 ± 0.25				
SCN5A + WT/MT SCN3B	8	−80.93 ± 4.72					4.56 ± 0.35				

The results are expressed as mean ± standard deviation. V_1/2_, voltage of half-maximal activation or inactivation; k, slope factor; WT, wild-type; MT, mutant type; WT/MT, co-expression of wild-type and mutant; WT-MT, comparison between wild-type and mutant groups; WT-WT/MT: comparison between wild-type group and co-expressing group; MT-WT/MT, comparison between mutant group and co-expressing group; *P*, statistical significance value.

**Table 3:** Steady-state recovery parameters of sodium channels in HEK 293 cells co-expressing SCN5A with either wild-type (WT), mutant (MT), or a combination of WT and MT SCN3B.

Group	*n*	τ (ms)				
		Value	*P*	*P*_WT-MT_	*P*_WT-WT/MT_	*P*_MT-WT/MT_
SCN5A + WT SCN3B	8	0.0042 ± 0.00081	0.59	0.57	0.78	0.94
SCN5A + MT SCN3B	8	0.0038 ± 0.00037				
SCN5A + WT/MT SCN3B	8	0.0040 ± 0.00071				

The results are expressed as mean ± standard deviation. V_1/2_, voltage of half-maximal activation or inactivation; τ, time constant of recovery; WT, wild-type; MT, mutant type; WT/MT, co-expression of wild-type and mutant; WT-MT, comparison between wild-type and mutant groups; WT-WT/MT, comparison between wild-type group and co-expressing group; MT-WT/MT, comparison between mutant group and co-expressing group; *P*, statistical significance value.

The steady-state activation curves were fitted using the Boltzmann equation, as depicted in Figure 4E. The half-maximal activation voltages (V_1/2_) for the wild-type, mutant, and co-expressed groups were −40.8 ± 7.5 mv, −40.1 ± 5.0 mv, and −40.6 ± 6.0 mv, respectively (*p* = 0.98), while the slope factors (k) were 5.4 ± 1.4, 4.7 ± 1.2, and 5.0 ± 1.3 (*p* = 0.648), as listed in Table 1.

**Figure 5:**
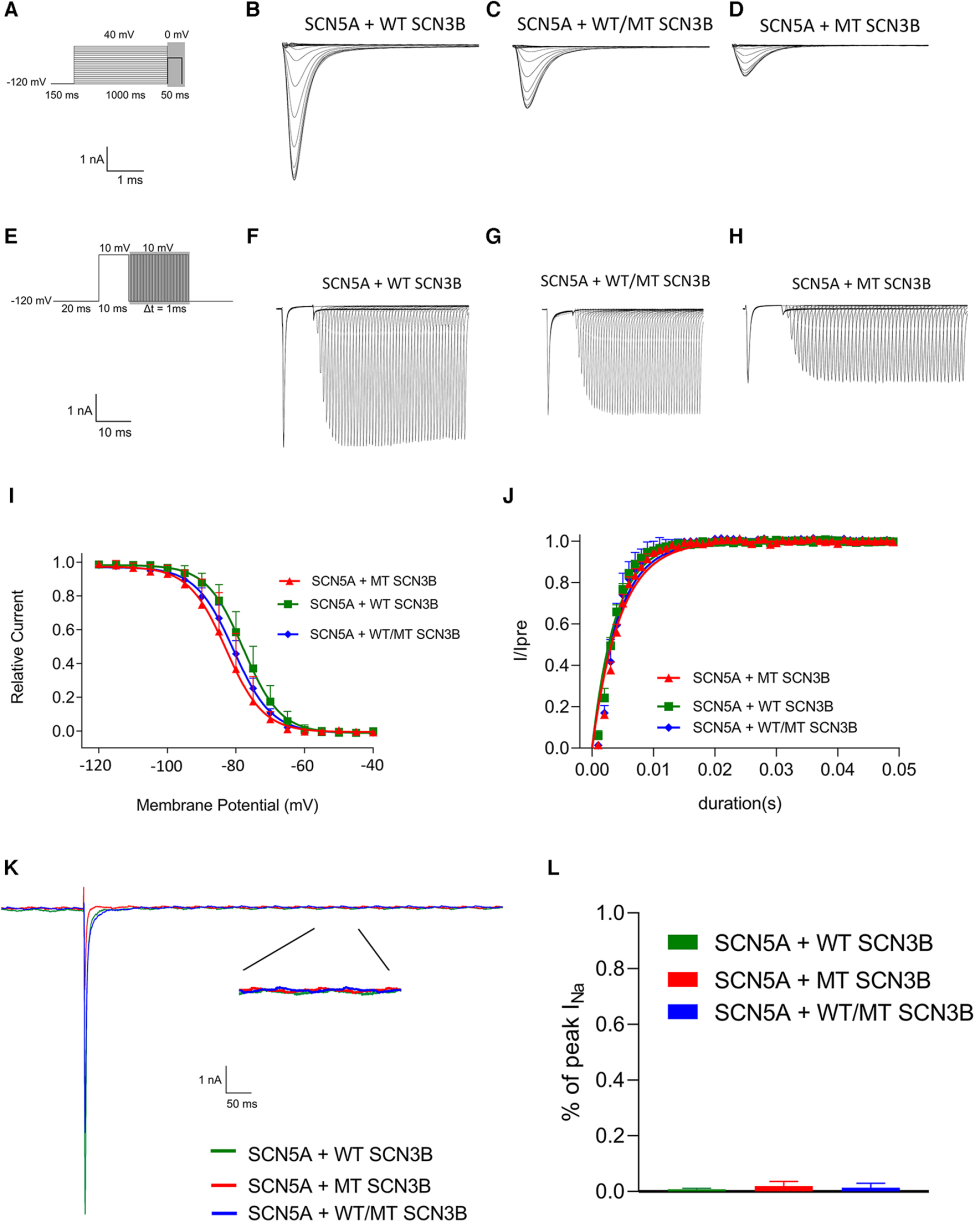
*SCN3B* P87L's influence on NaV1.5 inactivation, reactivation, and delayed sodium current. (**A**) Single-channel inactivation protocol is outlined in the upper panel, with the scale bar presented in the lower panel for reference. (**B–D**) Inactivation current traces for the wild-type group, the mutation group, and the group co-expressing both wild-type and mutation alleles. (**E**) The recovery stimulation protocol is depicted in the upper panel, with the corresponding scale bar in the lower panel. (**F–H**) Traces of recovery currents are shown for the wild-type group, the mutation group, and the group co-expressing both wild-type and mutation alleles. (**I**) The steady-state inactivation curve, modeled with a Boltzmann function, shows no significant difference in half-maximal inactivation voltage (V_1/2_) or slope factor (k) among the three groups (details provided in Table 2). (**J**) Recovery kinetics, fitted with an exponential function, reveal no significant differences in the time constant τ across the groups (refer to Table 3 for details). (**K**) Traces of the late sodium current for the wild-type group, the mutation group, and the group co-expressing both wild-type and mutation alleles are shown, including an enlarged view of the late INa current at the bottom. The late sodium currents are virtually identical across groups, with the scale provided below. (**L**) A bar graph representing the amplitude of the late INa current for each group indicates no significant difference between them (*p* = 0.23).

Inactivation of I_Na_ was analyzed based on protocols shown in the upper panel of Figure 5A, with all groups displaying I_Na_ inactivation currents (Figures 5B–D). Steady-state inactivation curves, represented in Figure 5I, were modeled using the Boltzmann equation. The corresponding semi-inactivation voltages (V_1/2)_ and slope factors (k) for the wild-type, mutant, and co-expressed groups are presented in Table 2, with no significant statistical differences observed (*p*-values of 0.08 and 0.25, respectively).

Figure 5E outlines the recovery pulse protocol used for HEK293 cells, with recovery curves demonstrated in Figures 5F–H and analyzed using an exponential equation (Figure 5J). No significant differences were found in the recovery time constant τ between the wild-type, mutant, and co-expression groups (*p* = 0.59, as indicated in Table 3).

Additionally, as shown in Figure 4K, the late sodium currents for the wild-type, mutant, and co-expressed groups were assessed. There was no statistically significant difference in late sodium current between these groups (*p* = 0.23, as shown in Figure 4K).

### Simulation of human cardiomyocytes action potential (AP)

**Figure 6:**
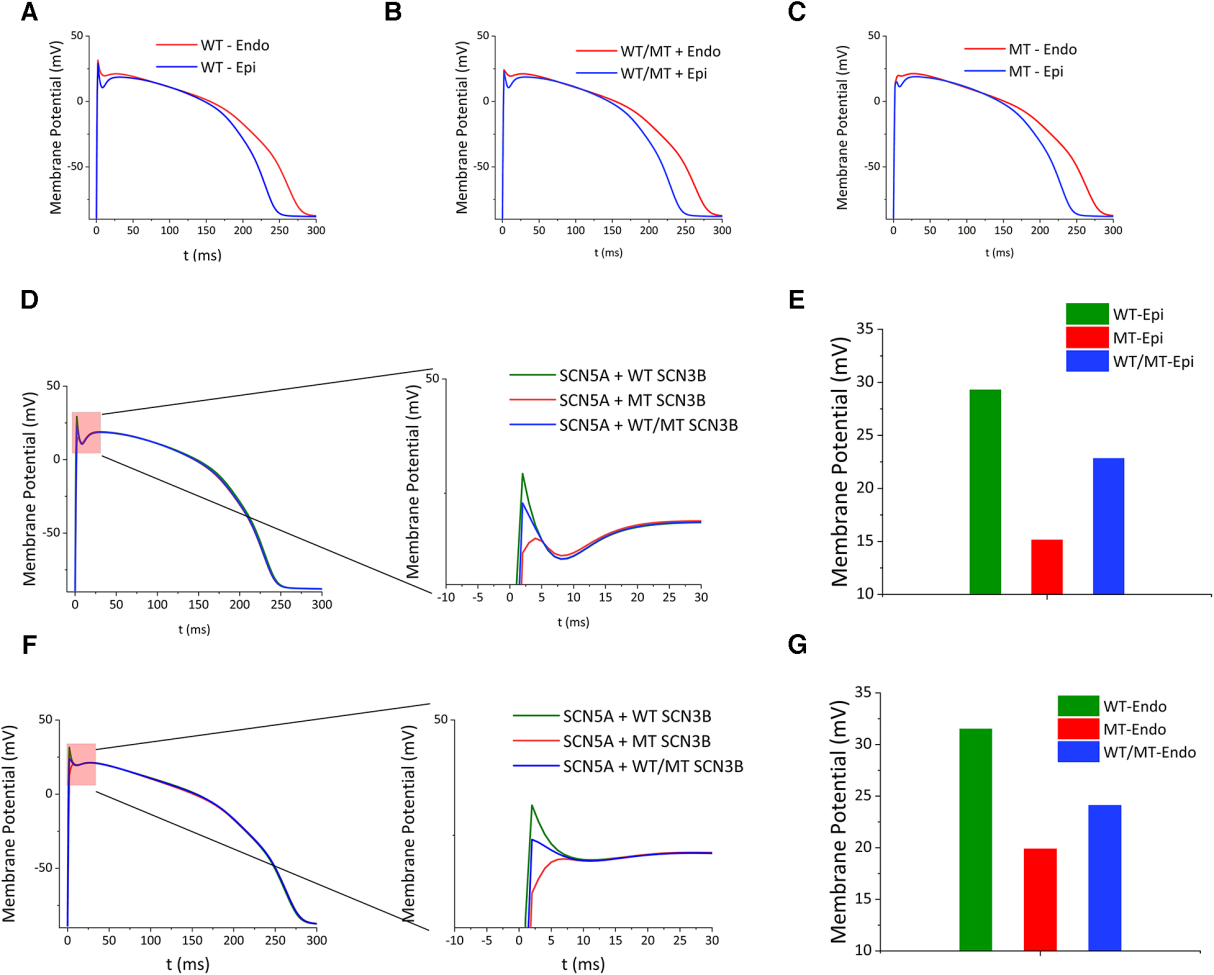
Impact of SCN3B P87l mutation on cardiac action potentials. (**A–C**) Simulated epicardial action potential waveforms for the wild-type group, the group co-expressing wild-type and mutant alleles, and the mutant group. (**D**) The left panel illustrates the simulated epicardial action potential waveforms for the wild-type, co-expression, and mutant groups. The right panel presents a zoomed-in view of the critical red segment for enhanced detail. (**E**) Bar graph showing the peak epicardial depolarization voltages for the wild-type group, the mutant group, and the group co-expressing wild-type and mutant alleles. (**F**) The left panel depicts simulations of endocardial action potential waveforms for the wild-type group, the group co-expressing wild-type and mutant alleles, and the mutant group. The right panel features a zoomed-in view of the designated red section. (**G**) Bar graph representing the peak endocardial depolarization voltages for the wild-type group, the mutant group, and the group co-expressing wild-type and mutant alleles.

To evaluate the impact of genetic variations on cardiac electrophysiology, simulations comparing wild-type, mutant, and co-expressed SCN3B groups were performed. These simulations focused on the action potentials within the epicardial and endocardial layers. Divergences in action potential morphology were noticeable among the wild-type, mutant, and co-expressed groups (Figures 6A–C). Specifically, the epicardial action potentials (Figure 6D) displayed maximum depolarization voltages of 29.29 mv for the wild-type, 15.14 mv for the mutant, and 22.84 mv for the co-expressed group (Figure 6E). Similarly, the endocardial action potentials (Figure 6F) revealed maximum depolarization voltages of 31.52 mv for the wild-type, 19.89 mv for the mutant, and 24.10 mv for the co-expressed group (Figure 6G).

## Discussion

The amino acid substitution of proline to leucine at position 87 within the Navβ3 subunit modifies the site's hydrophilicity. Patch clamp assays revealed a significant reduction in sodium channel activation currents due to this mutation; however, it appeared to leave other electrophysiological properties unaffected. Confocal microscopy analyses indicated a diminished presence of P87l Navβ3 in the cell membrane and an increased localization in the cytoplasm. Western blot analyses further suggested a reduction in the total cellular and membrane expression of both Navβ3 and Nav1.5, while cytoplasmic expression of SCN3B was elevated due to the P87l mutation. Computational modeling of cardiomyocytes demonstrated that the SCN3B P87l mutation could influence the morphology of action potentials in both the endocardial and epicardial layers and reduce their maximum depolarization voltages.

Cardiomyocyte fast sodium ion channel Nav1.5 comprises α and β subunits that perform regulatory functions. The α subunit of cardiac sodium channels is encoded by *SCN5A*. In cardiomyocytes, there are four regulatory subunits of sodium channels β1–β4 (encoded by *SCN1B*-*SCN4B*, respectively) (9). While *SCN5A* mutations account for 11%–28% of patients with BrS, the proportion of mutations in sodium channel regulatory subunits is only about 1% (4).

Recently, there are only two published reports regarding the relationship between *SCN3B* gene mutations and BrS, which involve the L10P (10) mutation observed in the American population and the V110l (11) mutation identified in the Japanese population. Nevertheless, it remains unclear whether *SCN3B* is also associated with BrS in patients of Chinese ethnicity. In our study, we have identified a novel *SCN3B* mutation in Chinese population with BrS. Electrophysiological investigations suggest that the *SCN3B* P87l mutation exerts its pathogenic effect by reducing the activation current of the channel.

The understanding of BrS pathogenesis in patients with an *SCN3B* mutation primarily relies on evaluating sodium channel expression through confocal microscopy. Prior work by Hu et al. inferred a decrease in *SCN3B* and *SCN5A* translocation to the cell membrane, as observed via this method, as a possible trigger to BrS (12). However, their use of spherical and non-adherent HEK293 cells could have distorted the experimental findings. The restriction of cell assessments with confocal microscopy can also produce biased interpretations of cellular functioning, an issue that was also observed in another *SCN3B*-associated BrS research (11). In order to address these limitations, we have incorporated Western blot analysis into our research methodology for examining cellular expression. The findings from our Western blot testing indicate the possibility of the *SCN3B* P87l protein reducing the transport of both *SCN3B* protein and *SCN5A* sodium ion channels to the cell membrane, potentially influencing the activation current density of Nav1.5.

The primary mechanism driving ventricular tachycardia and fibrillation in BrS is phase-2 reentry (13). This mechanism involves the flow of current from cardiomyocytes featuring an AP platform to those without, leading to partial re-excitation (14). Such phase-2 reentry can yield premature systole, potentially resulting in reentrant tachycardia or ventricular fibrillation. Hypothetically, factors such as a decrease in the I_Na_ or L-type I_Ca_ or an increase in I_to_, along with any other time-dependent potassium current, might provoke the aforementioned change (15). When the Na^+^ channel current is inhibited, thereby reducing the phase 0 amplitude, the presence of I_to_ can potentiate the dip in phase 1's lowest point as well as decrease the efficacy of I_Ca_, consequently resulting in the loss of the AP plateau.

Previous studies have reported SCN3B mutations associated with atrial fibrillation and idiopathic ventricular fibrillation. In a study by Olesen (16) that encompassed 192 unrelated patients with lone atrial fibrillation, three non-synonymous SCN3B mutations—R6K, L10P, and M161T—were identified. Notably, these genetic variants were not observed in a control cohort. Electrophysiological studies conducted on these mutations demonstrated a consequential reduction in sodium channel current. Significant is that the L10P mutation has been implicated as a pathogenic variant associated with BrS (12). Additionally, research has found an increased incidence of atrial fibrillation among BrS patients (17). The pathogenesis of atrial fibrillation in BrS is under thorough investigation, with current evidence suggesting a multifaceted interaction of inducible factors, underlying substrate vulnerability, and modulatory influences such as autonomic nervous function or inflammatory response (18). Valdivia et al. described a patient carrying the SCN3B V54G mutation who presented with idiopathic ventricular fibrillation (10). Functional analysis of this particular mutation revealed that its co-expression with Nav1.5 led to a considerable decrease in peak sodium current and created a positive shift in channel inactivation compared to wild-type channels. Remarkably, the patient's electrocardiogram exhibited epsilon waves, which are unusual. In an interesting parallel, the patient's mother, who was an asymptomatic carrier of the mutation, displayed J-point elevation on her electrocardiogram. This individual case points to a possible intersection between BrS and idiopathic ventricular fibrillation, with loss-of-function in I_Na_ being implicated in both conditions, as well as in the development of early repolarization patterns.

Given the prominent role of SCN3B and SCN5A in BrS, our study also examined the presence of paralogs for both genes, utilizing the resource available at http://www.ensembl.org/. We did not identify any paralogs for SCN3B. Detailed information on the paralogs of SCN5A is provided in Supplementary Table S9. None of these SCN5A paralogs are considered pathogenic, with the specific analytical process and reasoning documented in Supplementary Presentation S1.

Considering the critical pathological mutations of SCN5A in BrS, we conducted a literature review on the prognostic impact of SCN5A mutation within this condition. In one study encompassing 415 individuals diagnosed with BrS, 60 were identified as carrying SCN5A gene mutations, while 355 did not harbor these mutations. Brugada syndrome patients with SCN5A mutations exhibit more conduction abnormalities on ECG and have higher risk for cardiac events (19). Subsequent investigations on this patient demographic employed a heterologous expression system established in TS201 cells transfected with the mutant SCN5A gene, followed by the assessment of sodium currents using the patch-clamp technique (20). It was observed that 28.9% of patients with loss-of-function (LOF) sodium currents attributed to SCN5A mutations experienced lethal arrhythmic events, in contrast to none in the non-LOF group. This observation was echoed in another study following 40 BrS patients over a period of 2 years (21).

### The disadvantage of our research

A main limitation of our investigation is the use of the HEK293 cell model (22). This cell type displays significant differences from ventricular cardiomyocytes, given that human cardiomyocytes are highlighted by their high level of differentiation and exclusive expression of certain types of ion channels and cellular structural proteins. Consequently, the inferences made from the HEK293 cells cannot be fully generalized to human cardiomyocytes. Furthermore, our research utilized the Tusscher model to simulate the influences of *SCN3B* P87l on epicardial and endocardial action potentials. The electrophysiological traits of the simulated action potentials diverged notably from those characteristic of human cardiomyocytes (23).

## Conclusions

In summary, the P87l mutation in Navβ3 causes hydrophilicity changes and significantly reduces sodium channel currents as shown by patch clamp assays, without altering other cell electrophysiological properties. Confocal imaging reveals a redistribution of P87l Navβ3 from the membrane to the cytoplasm, and Western blot analysis indicates lowered expression of Navβ3 and Nav1.5 with increased cytoplasmic SCN3B. Computational modeling indicates altered action potential morphology and decreased depolarization voltages in myocardial layers. These insights deepen our understanding of SCN3B's role in BrS and its electrophysiological repercussions.

## Data Availability

The datasets generated during and/or analyzed during the current study are not publicly available due to the confidentiality agreement with the participating institution but are available from the corresponding author on reasonable request.
