# Supplementary material for: Identification of a novel Scn3b mutation in a Chinese Brugada syndrome pedigree: implications for Nav1.5 electrophysiological properties and intracellular distribution of Nav1.5 and Navβ3
Source: Front Cardiovasc Med. 2024 Feb 20;11:1320687. doi: 10.3389/fcvm.2024.1320687 (PMC10916001; doi:10.3389/fcvm.2024.1320687)
Supplement: Supplementary file 1 [file Table1.docx]

Supplementary Table 1. The details of these suspect pathogenic genes

| Gene (Chromosome Number) | Nucleotide Change (Exon Number) | Amino Acid Change (Variant Number) | Amino Acid Change (Variant Number) | Allele Frequency Distribution | Silicon Prediction |
| --- | --- | --- | --- | --- | --- |
| SCN3B  (chr11) | c.260C>T (Exon3) | p.P87L  (NM_018400) | N/A | N/A | Probable Damaging |
| CTNNA3  (chr10) | c.2378G>A (Exon 17) | p.G793E  (NM_013266) | rs188248522 | <0.005 | Probable Damaging |
| DPP6  (chr7) | c.58G>A (Exon 1) | p.A20T  (NM_130797) | rs2533731 | 0.00022 | Uncertain |
| PRKAG2  (chr7) | c.298G>A (Exon 3) | p.G100S  (NM_016203) | rs79474211 | <0.037 | Probable Damaging |
