# Supplementary material for: Identification of a novel Scn3b mutation in a Chinese Brugada syndrome pedigree: implications for Nav1.5 electrophysiological properties and intracellular distribution of Nav1.5 and Navβ3
Source: Front Cardiovasc Med. 2024 Feb 20;11:1320687. doi: 10.3389/fcvm.2024.1320687 (PMC10916001; doi:10.3389/fcvm.2024.1320687)
Supplement: Supplementary file 4 [file Table4.docx]

Supplementary Table 4. Rules for Combining Criteria to Classify Sequence Variants

|  | **Pathogenic** |  |
| --- | --- | --- |
|  | 1 | Very Strong (PVS1) AND  a. ≥1 Strong (PS1–PS4) OR  b. ≥2 Moderate (PM1–PM6) OR  c. 1 Moderate (PM1–PM6) and 1 Supporting (PP1–PP5) OR  d. ≥2 Supporting (PP1–PP5) |
|  | 2 | ≥2 Strong (PS1–PS4) OR |
|  | 3 | 1 Strong (PS1–PS4) AND  a. ≥3 Moderate (PM1–PM6) OR  b. 2 Moderate (PM1–PM6) AND ≥2 Supporting (PP1–PP5) OR  c. 1 Moderate (PM1–PM6) AND ≥4 Supporting (PP1–PP5) |
|  | **Likely Pathogenic** |  |
|  | 1 | 1 Very Strong (PVS1) AND 1 Moderate (PM1–PM6) OR |
|  | 2 | 1 Strong (PS1–PS4) AND 1–2 Moderate (PM1–PM6) OR |
|  | 3 | 1 Strong (PS1–PS4) AND ≥2 Supporting (PP1–PP5) OR |
|  | 4 | ≥3 Moderate (PM1–PM6) OR |
|  | 5 | 2 Moderate (PM1–PM6) AND ≥2 Supporting (PP1–PP5) OR |
|  | 6 | 1 Moderate (PM1–PM6) AND ≥4 Supporting (PP1–PP5) |
|  | **Benign** |  |
|  | 1 | 1 Stand-Alone (BA1) OR |
|  | 2 | ≥2 Strong (BS1–BS4) |
|  | **Likely Benign** |  |
|  | 1 | 1 Strong (BS1–BS4) and 1 Supporting (BP1–BP7) OR |
|  | 2 | ≥2 Supporting (BP1–BP7) |
