# Supplementary material for: Identification of a novel Scn3b mutation in a Chinese Brugada syndrome pedigree: implications for Nav1.5 electrophysiological properties and intracellular distribution of Nav1.5 and Navβ3
Source: Front Cardiovasc Med. 2024 Feb 20;11:1320687. doi: 10.3389/fcvm.2024.1320687 (PMC10916001; doi:10.3389/fcvm.2024.1320687)
Supplement: Supplementary file 5 [file Table5.docx]

**Supplementary Table 5. SCN3B P87L: Criteria for Classifying Pathogenic Variants**

|  |  | **Very strong evidence of pathogenicity** |
| --- | --- | --- |
| ✘ | PVS1 | Null variant (nonsense, frameshift, canonical +/−1 or 2 splice sites, initiation codon, single or multi-exon deletion) in a gene where loss of function (LOF) is a known mechanism of disease  Caveats:  • Beware of genes where LOF is not a known disease mechanism (e.g. GFAP, MYH7)  • Use caution interpreting LOF variants at the extreme 3’ end of a gene  • Use caution with splice variants that are predicted to lead to exon skipping but leave the remainder of the protein intact  • Use caution in the presence of multiple transcripts |
|  |  | **Strong evidence of pathogenicity** |
| ✘ | PS1 | Same amino acid change as a previously established pathogenic variant regardless of nucleotide change  Example:  Val->Leu caused by either G>C or G>T in the same codon.  Caveat:  Beware of changes that impact splicing rather than at the amino acid/protein level |
| ✘ | PS2 | De novo (both maternity and paternity confirmed) in a patient with the  disease and no family history  Note: Confirmation of paternity only is insufficient. Egg donation, surrogate motherhood, errors in embryo transfer, etc. can contribute to non-maternity |
| ✔ | PS3 | Well-established in vitro or in vivo functional studies supportive of a damaging effect on the gene or gene product  Note: Functional studies that have been validated and shown to be reproducible and robust in a clinical diagnostic laboratory setting are considered the most well-established |
| ✘ | PS4 | The prevalence of the variant in affected individuals is significantly increased compared to the prevalence in controls.  Note 1: Relative risk (RR) or odds ratio (OR), as obtained from case-control studies, is >5.0 and the confidence interval around the estimate of RR or OR does not include 1.0. See manuscript for detailed guidance.  Note 2: In instances of very rare variants where case-control studies may not reach statistical significance, the prior observation of the variant in multiple unrelated patients with the same phenotype, and its absence in controls, may be used as moderate level of evidence. |
|  |  | **Moderate evidence of pathogenicity** |
| ✘ | PM1 | Located in a mutational hot spot and/or critical and well-established functional domain (e.g. active site of an enzyme) without benign variation |
| ✔ | PM2 | Absent from controls (or at extremely low frequency if recessive) in Exome Sequencing Project, 1000 Genomes or ExAC  Caveat: Population data for indels may be poorly called by next generation sequencing |
| ✘ | PM3 | For recessive disorders, detected in trans with a pathogenic variant.  Note: This requires testing of parents (or offspring) to determine phase |
| ✘ | PM4 | Protein length changes due to in-frame deletions/insertions in a non-repeat region or stop-loss variants |
| ✘ | PM5 | Novel missense change at an amino acid residue where a different missense change determined to be pathogenic has been seen before  Example: Arg156His is pathogenic; now you observe Arg156CysCaveat: Beware of changes that impact splicing rather than at the amino acid/protein level |
| ✘ | PM6 | Assumed de novo, but without confirmation of paternity and maternity |
|  |  | **Supporting evidence of pathogenicity** |
| ✘ | PP1 | Co-segregation with disease in multiple affected family members in a gene definitively known to cause the disease  Note: May be used as stronger evidence with increasing segregation data |
| ✘ | PP2 | Missense variant in a gene that has a low rate of benign missense variation and where missense variants are a common mechanism of disease |
| ✔ | PP3 | Multiple lines of computational evidence support a deleterious effect on the gene or gene product (conservation, evolutionary, splicing impact, etc.)  Caveat: As many in silico algorithms use the same or very similar input for their predictions, each algorithm should not be counted as an independent criterion. PP3 can be used only once in any evaluation of a variant. |
| ✔ | PP4 | Patient’s phenotype or family history is highly specific for a disease with a single genetic etiology |
| ✘ | PP5 | Reputable source recently reports variant as pathogenic but the evidence is not available to the laboratory to perform an independent evaluation |
