# Supplementary material for: Identification of a novel Scn3b mutation in a Chinese Brugada syndrome pedigree: implications for Nav1.5 electrophysiological properties and intracellular distribution of Nav1.5 and Navβ3
Source: Front Cardiovasc Med. 2024 Feb 20;11:1320687. doi: 10.3389/fcvm.2024.1320687 (PMC10916001; doi:10.3389/fcvm.2024.1320687)
Supplement: Supplementary file 6 [file Table6.docx]

Supplementary Table 6. **SCN3B P87L:** Criteria for Classifying Benign Variants

|  |  | **Stand-Alone evidence of benign impact** |
| --- | --- | --- |
| ✘ | BA1 | Allele frequency is above 5% in Exome Sequencing Project, 1000 Genomes, or ExAC |
|  |  | **Strong evidence of benign impact** |
| ✘ | BS1 | Allele frequency is greater than expected for disorder |
| ✘ | BS2 | Observed in a healthy adult individual for a recessive (homozygous), dominant (heterozygous), or X-linked (hemizygous) disorder with full penetrance expected at an early age |
| ✘ | BS3 | Well-established in vitro or in vivo functional studies shows no damaging effect on protein function or splicing |
| ✘ | BS4 | Lack of segregation in affected members of a family  Caveat: The presence of phenocopies for common phenotypes (i.e. cancer, epilepsy) can mimic lack of segregation among affected individuals. Also, families may have more than one pathogenic variant contributing to an autosomal dominant disorder, further confounding an apparent lack of segregation. |
|  |  | **Supporting evidence of benign impact** |
| ✘ | BP1 | Missense variant in a gene for which primarily truncating variants are known to cause disease |
| ✘ | BP2 | Observed in trans with a pathogenic variant for a fully penetrant dominant gene/disorder; or observed in cis with a pathogenic variant in any inheritance pattern |
| ✘ | BP3 | In-frame deletions/insertions in a repetitive region without a known function |
| ✘ | BP4 | Multiple lines of computational evidence suggest no impact on gene or gene product (conservation, evolutionary, splicing impact, etc.)  Caveat: As many in silico algorithms use the same or very similar input for their predictions, each algorithm cannot be counted as an independent criterion. BP4 can be used only once in any evaluation of a variant. |
| ✘ | BP5 | Variant found in a case with an alternate molecular basis for disease |
| ✘ | BP6 | Reputable source recently reports variant as benign but the evidence is not available to the laboratory to perform an independent evaluation |
| ✘ | BP7 | A synonymous (silent) variant for which splicing prediction algorithms predict no impact to the splice consensus sequence nor the creation of anew splice site AND the nucleotide is not highly conserved |
