# Supplementary material for: Identification of a novel Scn3b mutation in a Chinese Brugada syndrome pedigree: implications for Nav1.5 electrophysiological properties and intracellular distribution of Nav1.5 and Navβ3
Source: Front Cardiovasc Med. 2024 Feb 20;11:1320687. doi: 10.3389/fcvm.2024.1320687 (PMC10916001; doi:10.3389/fcvm.2024.1320687)
Supplement: Supplementary file 7 [file Table8.docx]

| V (mV) | I_MT_ (pA/pF) | I_WT_ (pA/pF) | I_W/M_ (pA/pF) | *P* | *P*_WT-MT_ | *P*_MT-W/M_ | *P* _WT-W/M_ |
| --- | --- | --- | --- | --- | --- | --- | --- |
| -55 | -15.2064±8.752833 | -2.308625±2.019543 | -13.7481±10.4432 | 0.789 | 0.4969884 | 0.9907782 | 0.5745469 |
| -50 | -38.62422±15.2533 | -52.19062±11.75612 | -52.84878±19.60598 | 0.467 | 0.4969884 | 0.9907782 | 0.5745469 |
| -45 | -73.21101±20.11504 | -178.5563±33.14703 | -139.9144±40.40982 | 0.0893 | 0.0772562 | 0.3303379 | 0.6798166 |
| -40 | -102.5275±19.9472 | -302.2563±37.8644 | -208.8105±45.47908 | 0.00313 | 0.0021912 | 0.1173593 | 0.1835274 |
| -35 | -128.7814±19.04807 | -395.4438±41.75917 | -256.9017±43.22171 | 0.000176 | 0.0001119 | 0.0533483 | 0.0350179 |
| -30 | -142.7226±17.73377 | -435.5875±43.03622 | -279.4305±39.21288 | 3.51E-05 | 0.0000217 | 0.0308474 | 0.013076 |
| -25 | -150.8456±17.75415 | -450.8687±44.15494 | -288.7585±35.83517 | 1.90E-05 | 0.0000117 | 0.0257351 | 0.0085002 |
| -20 | -148.694±18.15694 | -440±43.85345 | -281.2215±33.64882 | 2.05E-05 | 0.0000127 | 0.0283875 | 0.0083154 |
| -15 | -144.502±16.40295 | -421.5625±43.26782 | -269.9485±29.99166 | 1.95E-05 | 0.0000121 | 0.0286033 | 0.0078525 |
| -10 | -133.7759±16.55849 | -397.6313±42.66182 | -250.0613±28.08862 | 2.60E-05 | 0.0000164 | 0.0380193 | 0.0078795 |
| -5 | -124.7904±15.35377 | -364.225±39.88715 | -231.4105±25.54463 | 3.48E-05 | 0.0000219 | 0.0404728 | 0.0098743 |
| 0 | -114.2629±14.04062 | -334.6438±40.44011 | -208.4±21.78683 | 6.25E-05 | 0.0000405 | 0.0638166 | 0.0110058 |
| 5 | -98.694±11.91204 | -296.7437±36.77101 | -181.9885±20.03545 | 6.90E-05 | 0.0000452 | 0.0714984 | 0.0107769 |
| 10 | -86.43738±10.26031 | -260.1562±35.82741 | 157.807±16.81495 | 0.000157 | 0.000106 | 0.1062235 | 0.0155111 |
| 15 | -71.9085±9.114537 | -221.6312±32.43681 | -130.6883±14.45453 | 0.000243 | 0.00017 | 0.1461262 | 0.0165033 |
| 20 | -57.08636±7.432337 | 183.5125±30.17548 | -104.1723±12.45951 | 0.000559 | 0.0004126 | 0.2204853 | 0.0222521 |
| 25 | -41.48484±6.447783 | -146.9269±28.61245 | -75.93475±10.87183 | 0.00162 | 0.0013318 | 0.3849675 | 0.0290123 |
| 30 | -29.19236±4.52448 | -109.5725±27.24486 | -52.4307±8.073438 | 0.00766 | 0.0069405 | 0.5916034 | 0.0598501 |
| 35 | -16.2066±3.009877 | -69.40587±23.27448 | -27.86812±6.843732 | 0.0356 | 0.0369983 | 0.8299551 | 0.1180852 |
| 40 | -4.013425±3.088339 | -38.03±21.017 | -5.574625±6.34524 | 0.13 | 0.1694514 | 0.9959083 | 0.1962398 |
| 45 | 5.946524±3.413729 | -14.20625±15.19723 | 9.7619±4.844754 | 0.179 | 0.3051901 | 0.9558651 | 0.1940628 |
| 50 | 15.51536±3.629189 | 8.98±7.989019 | 23.59555±4.06825 | 0.204 | 0.6904561 | 0.57084 | 0.1779435 |

Supplementary Table 8. Peak Current Density of SCN5A in HEK 293 Cells Co-Expressing with Either Wild-Type (WT), Mutant (MT), or a Combination of WT and MT SCN3B at Various Stimulus Voltages.

The results are expressed as mean ± standard error of the mean; WT: wild-type; MT: mutant type; W/M: co-expression of wild-type and mutant; V: stimulus voltage; I: maximum current density; WT-MT: comparison between wild-type and mutant groups; WT-W/M: comparison between wild-type and co-expressing groups; MT-W/M: comparison between mutant and co-expressing groups; P: statistical significance value.
