# Supplementary material for: Identification of a novel Scn3b mutation in a Chinese Brugada syndrome pedigree: implications for Nav1.5 electrophysiological properties and intracellular distribution of Nav1.5 and Navβ3
Source: Front Cardiovasc Med. 2024 Feb 20;11:1320687. doi: 10.3389/fcvm.2024.1320687 (PMC10916001; doi:10.3389/fcvm.2024.1320687)
Supplement: Supplementary file 9 [file Presentation1.pdf]

The primary criteria for SCN3B P87L assessment as Likely Pathogenic are: **one strong evidence (PS1-PS4) and one to two moderate evidence (PM1-PM6)**. Specifically, PS3 is supported by our cellular electrophysiological findings. Furthermore, the Moderate criterion (PM2) is fulfilled by the absence of the SCN3B P87L variant in control datasets as confirmed through various databases, including gnomAD (https://gnomad.broadinstitute.org/gene/ENSG00000166257?dataset=gnomad_r4), HGDP (https://www.internationalgenome.org/data-portal/data-collection/hgdp), 1KG (https://www.internationalgenome.org/), dbSNP (https://www.ncbi.nlm.nih.gov/snp/), ClinVar (https://www.ncbi.nlm.nih.gov/clinvar/), All of Us research program (https://databrowser.researchallofus.org/), and both GRCh38/hg38 (https://www.ncbi.nlm.nih.gov/datasets/genome/GCF_000001405.26/) and GRCh19/hg19 (https://www.ncbi.nlm.nih.gov/datasets/genome/GCF_000001405.13/) databases. Our inquiry indicates an absence of the SCN3B P87L variant in the general population, suggesting a low frequency of benign variants.

documentation

mutation t@sting

Prediction disease causing

Model: *simple_aae*, prob: 0.999999940356603 (explain)

hyperlink

Summary

amino acid sequence changed
protein features (might be) affected

analysed issue	analysis result
name of alteration	no title
alteration (phys. location)	chr11: 123513339G>A show variant in all transcripts IGV
HGNC symbol	SCN3B
Ensembl transcript ID	ENST00000392770
Genbank transcript ID	NM_018400
UniProt peptide	Q9NY72
alteration type	single base exchange
alteration region	CDS
DNA changes	c.260C>T<br>cDNA. 1063C>T<br>g. 12614C>T
AA changes	P87L Score: 98 explain score(s)
position(s) of altered AA<br>if AA alteration in CDS	87
frameshift	no
known variant	Variant was neither found in ExAC nor 1000G.<br>Search ExAC.
regulatory features	H3K27me3, Histone, Histone 3 Lysine 27 Tri-Methylation
phyloP / phastCons	see below
splice sites	no abrogation of potential splice sites
distance from splice site	41
Kozak consensus sequence altered?	N/A

phyloP / phastCons:

	PhyloP	PhastCons
(flanking)	0.943	1
	2. 148	1
(flanking)	5.93	1

explain score(s) and/or inspect your position(s) in in UCSC Genome Browser

conservation
protein level for non-synonymous changes

species	match	gene	aa	alignment
Human			87	E Y R N G H Q E V E S P F Q G R L Q W N G S K D
mutated	not conserved		87	E Y R N G H Q E V E S L F Q G R L Q W N G S K
Ptroglodytes	all identical	ENSPTRG00000004408	87	E Y R N G H Q E V E S P F Q G R L Q W N G S K
	all identical	ENSMMUG00000000343	87	E Y R N G H Q E V E S P F Q G R L Q W N G S K
	all	ENSFCAG00000000425	86	E Y R N G Q Q E V E S P F Q G R L Q W N G S K

Mmulatta

Fcatus

Mmusculus

	identical			
	not conserved	ENSGALG00000006490	87	E H R K M N H E F P S R F S G R I Q W N G S K
Trubripes	all identical	ENSTRUG00000014777	87	R F E D Y N LI E M D G P F K G R L F W N G T Q
Drerio	not conserved	ENSDARG00000062359	108	L Y D G E P Q EPKD V D E Q W K G R V T W T G S K
Dmelanogaster	no homologue			
Celegans	no homologue			
Xtropicalis	all identical	ENSXETG00000001239	87	E Y D G K P L E S K S P L Q G R L Q W T G S K

protein features	start (aa)	end (aa)	feature	details	
	23	159	TOPO_DOM	Extracellular (Potential).	lost
	32	154	DOMAIN	Ig-like C2-type.	lost

length of protein	normal
AA sequence altered	yes
position of stopcodon in wt / mu CDS	648 / 648
position (AA) of stopcodon in wt / mu AA sequence	216 / 216
position of stopcodon in wt / mu cDNA	1451 / 1451
poly(A) signal	N/A
conservation nucleotide level for all changes - no scoring up to now	N/A
position of start ATG in wt / mu cDNA	804 / 804
chromosome	11
strand	-1
last intron/exon boundary	1474
theoretical NMD boundary in CDS	620
length of CDS	648
coding sequence (CDS) position	260
cDNA position (for ins/del: last normal base / first normal base)	1063
gDNA position (for ins/del: last normal base / first normal base)	12614
chromosomal position (for ins/del: last normal base / first normal base)	123513339
original gDNA sequence snippet	CCACCAGGAGGTGGAGAGCCCCTTTCAGGGGCGCCTGCAGT
altered gDNA sequence snippet	CCACCAGGAGGTGGAGAGCCTCTTTCAGGGGCGCCTGCAGT
original cDNA sequence snippet	CCACCAGGAGGTGGAGAGCCCCTTTCAGGGGCGCCTGCAGT
altered cDNA sequence snippet	CCACCAGGAGGTGGAGAGCCTCTTTCAGGGGCGCCTGCAGT
wildtype AA sequence	MPAFNRLFPL ASLVLIYWVS VCFPVCVEVP SETEAVQGNP MKLRCISCMK REEVEATTVV<br>EWFYRPEGGK DFLIYEYRNG HQEVESPFQG RLQWNGSKDL QDVSITVLNV TLNDSGLYTC

	NVSREFEFEA HRPFVKTTRL IPLRVTEEAG EDFTSVVSEI MMYILLVFLT LWLLIEMIYC<br>YRKVSKAEEA AQENASDYLA IPSENKENSA VPVEE*
mutated AA sequence	MPAFNRLFPL ASLVLIYWVS VCFPVCVEVP SETEAVQGNP MKLRCISCMK REEVEATTVV<br>EWFYRPEGGK DFLIYEYRNG HQEVESLFQG RLQWNGSKDL QDVSITVLNV TLNDSGLYTC<br>NVSREFEFEA HRPFVKTTRL IPLRVTEEAG EDFTSVVSEI MMYILLVFLT LWLLIEMIYC<br>YRKVSKAEEA AQENASDYLA IPSENKENSA VPVEE*
speed	0. 16 s
	Report bugs / help us to improve MutationTaster!

All positions are in basepairs (bp) if not explicitly stated differently.

AA/ aa: amino acid; CDS: coding sequence; mu: mutated; NMD: nonsense- mediated mRNA decay; nt: nucleotide; wt: wildtype; TGP: 1000 Genomes Project

PolyPhen-2 prediction of functional effects of human nsSNPs

Home About Help Downloads

PolyPhen-2 report for Q9NY72 P87L

Query

Protein Acc	Position	AA1	AA2	Description
Q9NY72	87	P	L	Canonical; RecName: Full=Sodium channel subunit beta-3; Flags: Precursor; Length: 215

Results

Prediction/Confidence ***PolyPhen-2 v2.2.2r398***

HumDiv

This mutation is predicted to be **POSSIBLY DAMAGING** with a score of **0.770** (sensitivity: **0.85**; specificity: **0.92**)

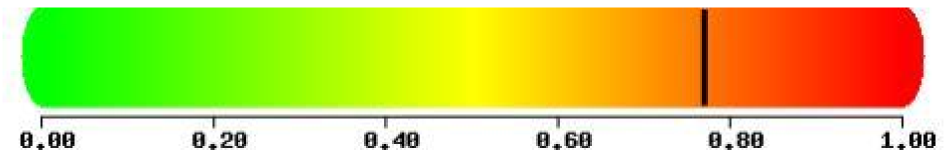

HumVar

Details

Multiple sequence alignment ***UCSC MultiZ46Way GRCh37/hg19 (08-Oct-2009)***

3D Visualization ***PDB/DSSP Snapshot 03-Jan-2012 (78304 Structures)***

Software & web support: ivan adzhubey Web design & development: biobyte solutions

JCVI J. CRAIG VENTER INSTITUTE PROVEAN

PROVEAN Result (Download)

PROVEAN Prediction - Job ID: 599981840421538

- **Query sequence** (fasta)
- **Supporting sequence set used for prediction**
  Number of sequences: **101** (fasta, E-values)
  Number of clusters: **30**
- **Score thresholds for prediction**
  (1) Default threshold is -2.5, that is:
  - Variants with a score equal to or below -2.5 are considered "deleterious,"
  - Variants with a score above -2.5 are considered "neutral."
  (2) How to use a more stringent threshold.

Variant	PROVEAN score	Prediction (cutoff= -2.5)
P87L	-3.363	Deleterious

- Submitted at 4:38:39 EDT, Saturday, May 22, 2021
- Started at 4:38:40 EDT, Saturday, May 22, 2021
- Finished at 4:38:40 EDT, Saturday, May 22, 2021

* The results are kept for 48 hours.

Mendelian Clinically Applicable Pathogenicity (M-CAP) Score

M-CAP is the first pathogenicity classifier for rare missense variants in the human genome that is tuned to the high sensitivity required in the clinic (see Table). By combining previous pathogenicity scores (including SIFT, Polyphen-2 and CADD) with novel features and a powerful model, we attain the best classifier at all thresholds, reducing a typical exome/genome rare (<1%) missense variant (VUS) list from 300 to 120, while never mistaking 95% of known pathogenic variants as benign. Further details can be found here (http://rdcu.be/lPtd).

3/25/2019: M-CAP score file with an interpretable sensitivity score can be found here (downloads/dat/mcap_v1_4.txt.gz)! Sensitivity score ≤ 0.95 is possibly pathogenic.

Method	Authors' Recommended Pathogenicity threshold	Misclassified known pathogenic variants
**SIFT**	< 0.05	38%
**Polyphen-2**	> 0.8	31%
**CADD**	> 20	26%
**MetaLR**	> 0.5	27%
**M-CAP**	> 0.025	5%

Score a variant

Enter the GRCh37/hg19 (http://genome.ucsc.edu/cgi-bin/hgGateway?db=hg19) coordinate for a missense variant to retrieve its M-CAP score.

GRCh37/hg19 | 11:123513339 | Go!

Demo!

GRCh37/hg19.11:123,513,339 (db=hg19&position=chr11%3A123513334-123513344&highlight=hg19.chr11%3A123513339-123513339&hgsid=945708001_LGsmzrOUjHkv8HvHJdhbmlWLI3Qz) Reference Allele G

Alt	M-CAP	95% TPR
A	0.031	Possibly Pathogenic
C	0.005	Likely Benign

M-CAP v1.0

Alt	M-CAP	95% TPR
T	0.022	Likely Benign

How to cite

Jagadeesh, K., Wenger, A., Berger, M., Guturu, H., Stenson, P., Cooper, D., Bernstein, J., and Bejerano, G. (2016). M-CAP eliminates a majority of variants with uncertain significance in clinical exomes at high sensitivity. Nature Genetics, 2016. 48 (12) 1581 DOI: 10.1038/ng.3703 (http://dx.doi.org/10.1038/ng.3703)

Download M-CAP Scores

M-CAP only scores rare missense variants: hg19, ENSEMBL 75 missense, ExAC v0.3
(
public/legacy/exacv1_downloads/release0.3) where no super population has minor allele frequency above 1%. If a missense variant has no M-CAP score, the M-CAP prediction should be assumed to be likely benign.

- ~~M-CAP v1.0 scores 10/24/2016 (downloads/dat/mcap_v1_1.txt.gz)~~ please do not use
- M-CAP v1.3 raw scores 10/30/2018 (downloads/dat/mcap_v1_3.txt.gz)
- M-CAP v1.4 raw and normalized scores 3/25/2019 (downloads/dat/mcap_v1_4.txt.gz) (v1.3 raw score of 0.025 converted to sensitivity score 0.95, to facilitate interpretation)

M-CAP source code

M-CAP public git repository can be found here (https://bitbucket.org/bejerano/mcap_public).
M-CAP train and test data can be found here (downloads/dat/).

Licensed under a Creative Commons Attribution-NonCommercial 4.0 International License (https://creativecommons.org/licenses/by-nc/4.0/). Please email bejerano@stanford.edu for commercial licensing inquiries.

Original bands of Western blot

GADPH

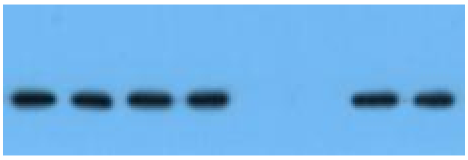

ATP1A2

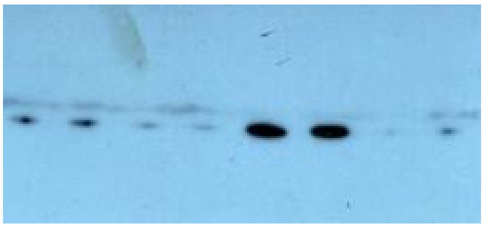

SCN5A

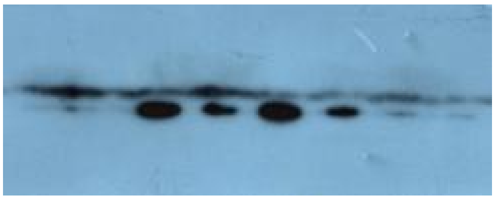

SCN3B

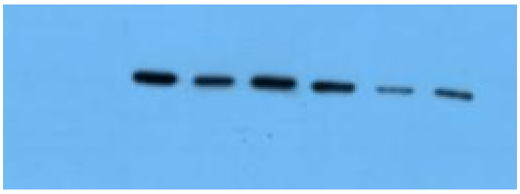

CTNNA3(G793E): Criteria for Classifying Pathogenic Variants

		**Very strong evidence of pathogenicity**
✘	PVS1	Null variant (nonsense, frameshift, canonical +/−1 or 2 splice sites, initiation codon, single or multi-exon deletion) in a gene where loss of function (LOF) is a known mechanism of disease<br>Caveats:<br>• Beware of genes where LOF is not a known disease mechanism (e.g. GFAP, MYH7)<br>• Use caution interpreting LOF variants at the extreme 3' end of a gene<br>• Use caution with splice variants that are predicted to lead to exon skipping but leave the remainder of the protein intact<br>• Use caution in the presence of multiple transcripts
		**Strong evidence of pathogenicity**
✘	PS1	Same amino acid change as a previously established pathogenic variant regardless of nucleotide change<br>Example:<br>Val->Leu caused by either G>C or G>T in the same codon.<br>Caveat:<br>Beware of changes that impact splicing rather than at the amino acid/protein level
✘	PS2	De novo (both maternity and paternity confirmed) in a patient with the disease and no family history<br>Note: Confirmation of paternity only is insufficient. Egg donation, surrogate motherhood, errors in embryo transfer, etc. can contribute to non-maternity
✘	PS3	Well-established in vitro or in vivo functional studies supportive of a damaging effect on the gene or gene product<br>Note: Functional studies that have been validated and shown to be reproducible and robust in a clinical diagnostic laboratory setting are considered the most well-established
✘	PS4	The prevalence of the variant in affected individuals is significantly increased compared to the prevalence in controls.<br>Note 1: Relative risk (RR) or odds ratio (OR), as obtained from case-control studies, is >5.0 and the confidence interval around the estimate of RR or OR does not include 1.0. See manuscript for detailed guidance.<br>Note 2: In instances of very rare variants where case-control studies may not reach statistical significance, the prior observation of the variant in multiple unrelated patients with the same phenotype, and its absence in controls, may be used as moderate level of evidence.
		**Moderate evidence of pathogenicity**
✘	PM1	Located in a mutational hot spot and/or critical and well-established functional domain (e.g. active site of an enzyme) without benign variation
✔	PM2	Absent from controls (or at extremely low frequency if recessive) in Exome Sequencing Project, 1000 Genomes or ExAC<br>Caveat: Population data for indels may be poorly called by next generation

		sequencing
✘	PM3	For recessive disorders, detected in trans with a pathogenic variant.<br>Note: This requires testing of parents (or offspring) to determine phase
✘	PM4	Protein length changes due to in-frame deletions/insertions in a non-repeat region or stop-loss variants
✘	PM5	Novel missense change at an amino acid residue where a different missense change determined to be pathogenic has been seen before<br>Example: Arg156His is pathogenic; now you observe Arg156CysCaveat: Beware of changes that impact splicing rather than at the amino acid/protein level
✘	PM6	Assumed de novo, but without confirmation of paternity and maternity
		**Supporting evidence of pathogenicity**
✘	PP1	Co-segregation with disease in multiple affected family members in a gene definitively known to cause the disease<br>Note: May be used as stronger evidence with increasing segregation data
✘	PP2	Missense variant in a gene that has a low rate of benign missense variation and where missense variants are a common mechanism of disease
✔	PP3	Multiple lines of computational evidence support a deleterious effect on the gene or gene product (conservation, evolutionary, splicing impact, etc.)<br>Caveat: As many in silico algorithms use the same or very similar input for their predictions, each algorithm should not be counted as an independent criterion. PP3 can be used only once in any evaluation of a variant.
✘	PP4	Patient’s phenotype or family history is highly specific for a disease with a single genetic etiology
✘	PP5	Reputable source recently reports variant as pathogenic but the evidence is not available to the laboratory to perform an independent evaluation

CTNNA3(G793E): Criteria for Classifying Benign Variants

		**Stand-Alone evidence of benign impact**
✘	BA1	Allele frequency is above 5% in Exome Sequencing Project, 1000 Genomes, or ExAC
		**Strong evidence of benign impact**
✘	BS1	Allele frequency is greater than expected for disorder
✘	BS2	Observed in a healthy adult individual for a recessive (homozygous), dominant (heterozygous), or X-linked (hemizygous) disorder with full penetrance expected at an early age
✘	BS3	Well-established in vitro or in vivo functional studies shows no damaging effect on protein function or splicing
✔	BS4	Lack of segregation in affected members of a family<br>Caveat: The presence of phenocopies for common phenotypes (i.e. cancer,

		epilepsy) can mimic lack of segregation among affected individuals. Also, families may have more than one pathogenic variant contributing to an autosomal dominant disorder, further confounding an apparent lack of segregation.
		**Supporting evidence of benign impact**
✘	BP1	Missense variant in a gene for which primarily truncating variants are known to cause disease
✘	BP2	Observed in trans with a pathogenic variant for a fully penetrant dominant gene/disorder; or observed in cis with a pathogenic variant in any inheritance pattern
✘	BP3	In-frame deletions/insertions in a repetitive region without a known function
✔	BP4	Multiple lines of computational evidence suggest no impact on gene or gene product (conservation, evolutionary, splicing impact, etc.)<br>Caveat: As many in silico algorithms use the same or very similar input for their predictions, each algorithm cannot be counted as an independent criterion. BP4 can be used only once in any evaluation of a variant.
✘	BP5	Variant found in a case with an alternate molecular basis for disease
✔	BP6	Reputable source recently reports variant as benign but the evidence is not available to the laboratory to perform an independent evaluation
✘	BP7	A synonymous (silent) variant for which splicing prediction algorithms predict no impact to the splice consensus sequence nor the creation of anew splice site AND the nucleotide is not highly conserved

CTNNA3 (G793E): Rules for Combining Criteria to Classify Sequence Variants

	**Pathogenic**	
✘	1	Very Strong (PVS1) AND<br>a. ≥1 Strong (PS1–PS4) OR<br>b. ≥2 Moderate (PM1–PM6) OR<br>c. 1 Moderate (PM1–PM6) and 1 Supporting (PP1–PP5) OR<br>d. ≥2 Supporting (PP1–PP5)
	2	≥2 Strong (PS1–PS4) OR
✘	3	1 Strong (PS1–PS4) AND<br>a. ≥3 Moderate (PM1–PM6) OR<br>b. 2 Moderate (PM1–PM6) AND ≥2 Supporting (PP1–PP5) OR<br>c. 1 Moderate (PM1–PM6) AND ≥4 Supporting (PP1–PP5)
	**Likely Pathogenic**	
✘	1	1 Very Strong (PVS1) AND 1 Moderate (PM1–PM6) OR
✘	2	1 Strong (PS1–PS4) AND 1–2 Moderate (PM1–PM6) OR
✘	3	1 Strong (PS1–PS4) AND ≥2 Supporting (PP1–PP5) OR
✘	4	≥3 Moderate (PM1–PM6) OR
✘	5	2 Moderate (PM1–PM6) AND ≥2 Supporting (PP1–PP5) OR

✘	6	1 Moderate (PM1–PM6) AND ≥4 Supporting (PP1–PP5)
	**Benign**	
✘	1	1 Stand-Alone (BA1) OR
✘	2	≥2 Strong (BS1–BS4)
	**Likely Benign**	
✔	1	1 Strong (BS1–BS4) and 1 Supporting (BP1–BP7) OR
✔	2	≥2 Supporting (BP1–BP7)

DPP6 (A20T): Criteria for Classifying Pathogenic Variants

		**Very strong evidence of pathogenicity**
✘	PVS1	Null variant (nonsense, frameshift, canonical +/−1 or 2 splice sites, initiation codon, single or multi-exon deletion) in a gene where loss of function (LOF) is a known mechanism of disease<br>Caveats:<br>• Beware of genes where LOF is not a known disease mechanism (e.g. GFAP, MYH7)<br>• Use caution interpreting LOF variants at the extreme 3' end of a gene<br>• Use caution with splice variants that are predicted to lead to exon skipping but leave the remainder of the protein intact<br>• Use caution in the presence of multiple transcripts
		**Strong evidence of pathogenicity**
✘	PS1	Same amino acid change as a previously established pathogenic variant regardless of nucleotide change<br>Example:<br>Val->Leu caused by either G>C or G>T in the same codon.<br>Caveat:<br>Beware of changes that impact splicing rather than at the amino acid/protein level
✘	PS2	De novo (both maternity and paternity confirmed) in a patient with the disease and no family history<br>Note: Confirmation of paternity only is insufficient. Egg donation, surrogate motherhood, errors in embryo transfer, etc. can contribute to non-maternity
✘	PS3	Well-established in vitro or in vivo functional studies supportive of a damaging effect on the gene or gene product<br>Note: Functional studies that have been validated and shown to be reproducible and robust in a clinical diagnostic laboratory setting are considered the most well-established
✘	PS4	The prevalence of the variant in affected individuals is significantly increased compared to the prevalence in controls.<br>Note 1: Relative risk (RR) or odds ratio (OR), as obtained from case-control studies, is >5.0 and the confidence interval around the estimate of RR or OR does not include 1.0. See manuscript for detailed guidance.<br>Note 2: In instances of very rare variants where case-control studies may not reach

		statistical significance, the prior observation of the variant in multiple unrelated patients with the same phenotype, and its absence in controls, may be used as moderate level of evidence.
		**Moderate evidence of pathogenicity**
✘	PM1	Located in a mutational hot spot and/or critical and well-established functional domain (e.g. active site of an enzyme) without benign variation
✔	PM2	Absent from controls (or at extremely low frequency if recessive) in Exome Sequencing Project, 1000 Genomes or ExAC<br>Caveat: Population data for indels may be poorly called by next generation sequencing
✘	PM3	For recessive disorders, detected in trans with a pathogenic variant.<br>Note: This requires testing of parents (or offspring) to determine phase
✘	PM4	Protein length changes due to in-frame deletions/insertions in a non-repeat region or stop-loss variants
✘	PM5	Novel missense change at an amino acid residue where a different missense change determined to be pathogenic has been seen before<br>Example: Arg156His is pathogenic; now you observe Arg156CysCaveat: Beware of changes that impact splicing rather than at the amino acid/protein level
✘	PM6	Assumed de novo, but without confirmation of paternity and maternity
		**Supporting evidence of pathogenicity**
✘	PP1	Co-segregation with disease in multiple affected family members in a gene definitively known to cause the disease<br>Note: May be used as stronger evidence with increasing segregation data
✘	PP2	Missense variant in a gene that has a low rate of benign missense variation and where missense variants are a common mechanism of disease
✘	PP3	Multiple lines of computational evidence support a deleterious effect on the gene or gene product (conservation, evolutionary, splicing impact, etc.)<br>Caveat: As many in silico algorithms use the same or very similar input for their predictions, each algorithm should not be counted as an independent criterion. PP3 can be used only once in any evaluation of a variant.
✘	PP4	Patient's phenotype or family history is highly specific for a disease with a single genetic etiology
✘	PP5	Reputable source recently reports variant as pathogenic but the evidence is not available to the laboratory to perform an independent evaluation

DPP6 (A20T): Criteria for Classifying Benign Variants

		**Stand-Alone evidence of benign impact**
✘	BA1	Allele frequency is above 5% in Exome Sequencing Project, 1000 Genomes, or ExAC

		**Strong evidence of benign impact**
✘	BS1	Allele frequency is greater than expected for disorder
✘	BS2	Observed in a healthy adult individual for a recessive (homozygous), dominant (heterozygous), or X-linked (hemizygous) disorder with full penetrance expected at an early age
✘	BS3	Well-established in vitro or in vivo functional studies shows no damaging effect on protein function or splicing
✔	BS4	Lack of segregation in affected members of a family<br>Caveat: The presence of phenocopies for common phenotypes (i.e. cancer, epilepsy) can mimic lack of segregation among affected individuals. Also, families may have more than one pathogenic variant contributing to an autosomal dominant disorder, further confounding an apparent lack of segregation.
		**Supporting evidence of benign impact**
✘	BP1	Missense variant in a gene for which primarily truncating variants are known to cause disease
✘	BP2	Observed in trans with a pathogenic variant for a fully penetrant dominant gene/disorder; or observed in cis with a pathogenic variant in any inheritance pattern
✘	BP3	In-frame deletions/insertions in a repetitive region without a known function
✔	BP4	Multiple lines of computational evidence suggest no impact on gene or gene product (conservation, evolutionary, splicing impact, etc.)<br>Caveat: As many in silico algorithms use the same or very similar input for their predictions, each algorithm cannot be counted as an independent criterion. BP4 can be used only once in any evaluation of a variant.
✘	BP5	Variant found in a case with an alternate molecular basis for disease
✘	BP6	Reputable source recently reports variant as benign but the evidence is not available to the laboratory to perform an independent evaluation
✘	BP7	A synonymous (silent) variant for which splicing prediction algorithms predict no impact to the splice consensus sequence nor the creation of anew splice site AND the nucleotide is not highly conserved

DPP6 (A20T): Rules for Combining Criteria to Classify Sequence Variants

	**Pathogenic**	
✘	1	Very Strong (PVS1) AND<br>a. ≥1 Strong (PS1–PS4) OR<br>b. ≥2 Moderate (PM1–PM6) OR<br>c. 1 Moderate (PM1–PM6) and 1 Supporting (PP1–PP5) OR<br>d. ≥2 Supporting (PP1–PP5)
✘	2	≥2 Strong (PS1–PS4) OR
✘	3	1 Strong (PS1–PS4) AND<br>a. ≥3 Moderate (PM1–PM6) OR

		b. 2 Moderate (PM1–PM6) AND ≥2 Supporting (PP1–PP5) OR<br>c. 1 Moderate (PM1–PM6) AND ≥4 Supporting (PP1–PP5)
	**Likely Pathogenic**	
✘	1	1 Very Strong (PVS1) AND 1 Moderate (PM1–PM6) OR
✘	2	1 Strong (PS1–PS4) AND 1–2 Moderate (PM1–PM6) OR
✘	3	1 Strong (PS1–PS4) AND ≥2 Supporting (PP1–PP5) OR
✘	4	≥3 Moderate (PM1–PM6) OR
✘	5	2 Moderate (PM1–PM6) AND ≥2 Supporting (PP1–PP5) OR
✘	6	1 Moderate (PM1–PM6) AND ≥4 Supporting (PP1–PP5)
	**Benign**	
✘	1	1 Stand-Alone (BA1) OR
✘	2	≥2 Strong (BS1–BS4)
	**Likely Benign**	
✔	1	1 Strong (BS1–BS4) and 1 Supporting (BP1–BP7) OR
✘	2	≥2 Supporting (BP1–BP7)

PRKAG2 (G100S): Criteria for Classifying Pathogenic Variants

		**Very strong evidence of pathogenicity**
✘	PVS1	Null variant (nonsense, frameshift, canonical +/−1 or 2 splice sites, initiation codon, single or multi-exon deletion) in a gene where loss of function (LOF) is a known mechanism of disease<br>Caveats:<br>• Beware of genes where LOF is not a known disease mechanism (e.g. GFAP, MYH7)<br>• Use caution interpreting LOF variants at the extreme 3' end of a gene<br>• Use caution with splice variants that are predicted to lead to exon skipping but leave the remainder of the protein intact<br>• Use caution in the presence of multiple transcripts
		**Strong evidence of pathogenicity**
✘	PS1	Same amino acid change as a previously established pathogenic variant regardless of nucleotide change<br>Example:<br>Val->Leu caused by either G>C or G>T in the same codon.<br>Caveat:<br>Beware of changes that impact splicing rather than at the amino acid/protein level
✘	PS2	De novo (both maternity and paternity confirmed) in a patient with the disease and no family history<br>Note: Confirmation of paternity only is insufficient. Egg donation, surrogate motherhood, errors in embryo transfer, etc. can contribute to non-maternity
✘	PS3	Well-established in vitro or in vivo functional studies supportive of a damaging effect on the gene or gene product

		Note: Functional studies that have been validated and shown to be reproducible and robust in a clinical diagnostic laboratory setting are considered the most well-established
✘	PS4	The prevalence of the variant in affected individuals is significantly increased compared to the prevalence in controls.<br>Note 1: Relative risk (RR) or odds ratio (OR), as obtained from case-control studies, is >5.0 and the confidence interval around the estimate of RR or OR does not include 1.0. See manuscript for detailed guidance.<br>Note 2: In instances of very rare variants where case-control studies may not reach statistical significance, the prior observation of the variant in multiple unrelated patients with the same phenotype, and its absence in controls, may be used as moderate level of evidence.
		**Moderate evidence of pathogenicity**
✘	PM1	Located in a mutational hot spot and/or critical and well-established functional domain (e.g. active site of an enzyme) without benign variation
✘	PM2	Absent from controls (or at extremely low frequency if recessive) in Exome Sequencing Project, 1000 Genomes or ExAC<br>Caveat: Population data for indels may be poorly called by next generation sequencing
✘	PM3	For recessive disorders, detected in trans with a pathogenic variant.<br>Note: This requires testing of parents (or offspring) to determine phase
✘	PM4	Protein length changes due to in-frame deletions/insertions in a non-repeat region or stop-loss variants
✘	PM5	Novel missense change at an amino acid residue where a different missense change determined to be pathogenic has been seen before<br>Example: Arg156His is pathogenic; now you observe Arg156CysCaveat: Beware of changes that impact splicing rather than at the amino acid/protein level
✘	PM6	Assumed de novo, but without confirmation of paternity and maternity
		**Supporting evidence of pathogenicity**
✘	PP1	Co-segregation with disease in multiple affected family members in a gene definitively known to cause the disease<br>Note: May be used as stronger evidence with increasing segregation data
✘	PP2	Missense variant in a gene that has a low rate of benign missense variation and where missense variants are a common mechanism of disease
✘	PP3	Multiple lines of computational evidence support a deleterious effect on the gene or gene product (conservation, evolutionary, splicing impact, etc.)<br>Caveat: As many in silico algorithms use the same or very similar input for their predictions, each algorithm should not be counted as an independent criterion. PP3 can be used only once in any evaluation of a variant.
✘	PP4	Patient’s phenotype or family history is highly specific for a disease with a single genetic etiology
✘	PP5	Reputable source recently reports variant as pathogenic but the evidence is not available to the laboratory to perform an independent evaluation

PRKAG2 (G100S): Criteria for Classifying Benign Variants

		**Stand-Alone evidence of benign impact**
✔	BA1	Allele frequency is above 5% in Exome Sequencing Project, 1000 Genomes, or ExAC
		**Strong evidence of benign impact**
✔	BS1	Allele frequency is greater than expected for disorder
✘	BS2	Observed in a healthy adult individual for a recessive (homozygous), dominant (heterozygous), or X-linked (hemizygous) disorder with full penetrance expected at an early age
✘	BS3	Well-established in vitro or in vivo functional studies shows no damaging effect on protein function or splicing
✔	BS4	Lack of segregation in affected members of a family<br>Caveat: The presence of phenocopies for common phenotypes (i.e. cancer, epilepsy) can mimic lack of segregation among affected individuals. Also, families may have more than one pathogenic variant contributing to an autosomal dominant disorder, further confounding an apparent lack of segregation.
		**Supporting evidence of benign impact**
✘	BP1	Missense variant in a gene for which primarily truncating variants are known to cause disease
✘	BP2	Observed in trans with a pathogenic variant for a fully penetrant dominant gene/disorder; or observed in cis with a pathogenic variant in any inheritance pattern
✘	BP3	In-frame deletions/insertions in a repetitive region without a known function
✔	BP4	Multiple lines of computational evidence suggest no impact on gene or gene product (conservation, evolutionary, splicing impact, etc.)<br>Caveat: As many in silico algorithms use the same or very similar input for their predictions, each algorithm cannot be counted as an independent criterion. BP4 can be used only once in any evaluation of a variant.
✘	BP5	Variant found in a case with an alternate molecular basis for disease
✘	BP6	Reputable source recently reports variant as benign but the evidence is not available to the laboratory to perform an independent evaluation
✘	BP7	A synonymous (silent) variant for which splicing prediction algorithms predict no impact to the splice consensus sequence nor the creation of anew splice site AND the nucleotide is not highly conserved

PRKAG2 (G100S): Rules for Combining Criteria to Classify Sequence Variants

	Pathogenic	
✘	1	Very Strong (PVS1) AND<br>a. ≥1 Strong (PS1–PS4) OR<br>b. ≥2 Moderate (PM1–PM6) OR<br>c. 1 Moderate (PM1–PM6) and 1 Supporting (PP1–PP5) OR<br>d. ≥2 Supporting (PP1–PP5)
✘	2	≥2 Strong (PS1–PS4) OR
✘	3	1 Strong (PS1–PS4) AND<br>a. ≥3 Moderate (PM1–PM6) OR<br>b. 2 Moderate (PM1–PM6) AND ≥2 Supporting (PP1–PP5) OR<br>c. 1 Moderate (PM1–PM6) AND ≥4 Supporting (PP1–PP5)
	**Likely Pathogenic**	
✘	1	1 Very Strong (PVS1) AND 1 Moderate (PM1–PM6) OR
✘	2	1 Strong (PS1–PS4) AND 1–2 Moderate (PM1–PM6) OR
✘	3	1 Strong (PS1–PS4) AND ≥2 Supporting (PP1–PP5) OR
✘	4	≥3 Moderate (PM1–PM6) OR
✘	5	2 Moderate (PM1–PM6) AND ≥2 Supporting (PP1–PP5) OR
✘	6	1 Moderate (PM1–PM6) AND ≥4 Supporting (PP1–PP5)
	**Benign**	
✔	1	1 Stand-Alone (BA1) OR
✘	2	≥2 Strong (BS1–BS4)
	**Likely Benign**	
✔	1	1 Strong (BS1–BS4) and 1 Supporting (BP1–BP7) OR
✘	2	≥2 Supporting (BP1–BP7)

Given the fact that 20% of patients with Brugada syndrome have mutations in the SCN5A gene, we fully recognize the significance of SCN5A gene mutations and consequently searched for its paralogs. The identified paralogs of SCN5A include CACNA1E, CACNA1F, CACNA1G, CACNA1H, CACNA1I, CACNA1S, CATSPER1, CATSPER2, CATSPER3, CATSPER4, NALCN, SCN10A, SCN11A, SCN1A, SCN2A, SCN3A, SCN4A, SCN7A, SCN8A, SCN9A, TPCN1, and TPCN2.

We screened the full genome sequencing data of the proband for mutations in these identified paralogs, with the results presented in Supplementary material 4. The screen revealed that, with the exception of a mutation in the SCN4A gene (ENST00000578147:exon24:c.C5277G:p.D1759E), all other paralog mutations are present with a frequency greater than 5% in the general population. Based on the Standards and guidelines for the interpretation of sequence variants by the American College of Medical Genetics and Genomics and the Association for Molecular Pathology (PMCID: PMC4544753), an allele frequency above 5% in Exome Sequencing Project, 1000 Genomes, or ExAC is considered benign. Thus, mutations other than SCN4A are deemed benign.

Subsequent investigation into the tissue-specific expression of SCN4A using The Human Protein Atlas (https://www.proteinatlas.org/ENSG00000007314-SCN4A/tissue#rna_expression) revealed substantial expression in skeletal muscle, the tongue, breast, and adipose tissue, but negligible expression in cardiac tissue. Therefore, we consider the identified SCN4A mutation unlikely to be pathogenic in our patient.

Based on the results detailed above, we conclude that the SCN5A paralogs are not the pathogenic variants in this patient with Brugada syndrome. We have incorporated the relevant content into the revised version of the manuscript accordingly.
